# Genome-Wide Association Study Reveals a Genetic Mechanism of Salt Tolerance Germinability in Rice (*Oryza sativa* L.)

**DOI:** 10.3389/fpls.2022.934515

**Published:** 2022-07-15

**Authors:** Caijing Li, Changsheng Lu, Baoli Zou, Mengmeng Yang, Guangliang Wu, Peng Wang, Qin Cheng, Yanning Wang, Qi Zhong, Shiying Huang, Tao Huang, Haohua He, Jianmin Bian

**Affiliations:** ^1^Key Laboratory of Crop Physiology, Ecology and Genetic Breeding, Ministry of Education, Nanchang, China; ^2^Key Laboratory of Crop Physiology, Ecology and Genetic Breeding, Nanchang, China

**Keywords:** salt tolerance germinability, rice accessions, GWAS, QTLs, haplotype analysis

## Abstract

Salt stress is one of the factors that limits rice production, and an important task for researchers is to cultivate rice with strong salt tolerance. In this study, 211 rice accessions were used to determine salt tolerance germinability (STG) indices and conduct a genome-wide association study (GWAS) using 36,727 SNPs. The relative germination energy (RGE), relative germination index (RGI), relative vigor index (RVI), relative mean germination time (RMGT), relative shoot length (RSL), and relative root length (RRL) were used to determine the STG indices in rice. A total of 43 QTLs, including 15 for the RGE, 6 for the RGI, 7 for the RVI, 3 for the RMGT, 1 for the RSL, and 11 for the RRL, were identified on nine chromosome regions under 60 and 100 mM NaCl conditions. For these STG-related QTLs, 18 QTLs were co-localized with previous studies, and some characterized salt-tolerance genes, such as *OsCOIN, OsHsp17.0*, and *OsDREB2A*, are located in these QTL candidates. Among the 25 novel QTLs, *qRGE60-1-2* co-localized with *qRGI60-1-1* on chromosome 1, and *qRGE60-3-1* and *qRVI60-3-1* co-localized on chromosome 3. According to the RNA-seq database, 16 genes, including nine for *qRGE60-1-2* (*qRGI60-1-1*) and seven for *qRGE60-3-1* (*qRVI60-3-1*), were found to show significant differences in their expression levels between the control and salt treatments. Furthermore, the expression patterns of these differentially expressed genes were analyzed, and nine genes (five for *qRGE60-1-2* and four for *qRGE60-3-1*) were highly expressed in embryos at the germination stage. Haplotype analysis of these nine genes showed that the rice varieties with elite haplotypes in the *LOC_Os03g13560, LOC_Os03g13840*, and *LOC_Os03g14180* genes had high STG. GWAS validated the known genes underlying salt tolerance and identified novel loci that could enrich the current gene pool related to salt tolerance. The resources with high STG and significant loci identified in this study are potentially useful in breeding for salt tolerance.

## Introduction

As the world's population continues to increase, researchers predict that food production must increase by at least 70% over the next 40 years to meet demand (Tilman et al., 2011). Rice is one of the world's most important food crops and feeds more than half of the world's population. Therefore, improving rice yields under conditions of limited arable land is an urgent problem for researchers to solve. The traditional rice transplanting mode is time-consuming and labor-intensive and has been gradually replaced in recent years by a direct seeding mode in many areas (Wang et al., 2011); at the same time, this development also means higher requirements for the germination ability of rice seeds in harsh environments.

Salt stress is one of the main factors that limits direct seeding of rice. Salinity alters the osmotic potential of the germination medium, enzyme activities in cells, and destroys cell structures so that seeds cannot germinate normally and establish stable stands of seedlings, which finally affects rice yields (Koyro, 2002; Khan and Weber, 2006; Gomes-Filho et al., 2008). Therefore, unraveling the genetic architecture for salt tolerance germinability and cultivating rice varieties with high STG are very important in accomplishing direct rice seeding.

To our knowledge, only a few genes have been reported to be involved in STG. Of those, OsHsp17.0 encodes heat shock proteins (Hsps), and its overexpression in plants demonstrated higher germination abilities than those of wild-type (WT) plants when subjected to NaCl (Zou et al., 2012). The basic helix-loop-helix (bHLH) transcription factor, OsbHLH035, is a salt-induced gene, and Osbhlh035 mutants exhibit delayed seed germination, especially under salt stress conditions (Chen et al., 2018). Under salt stress, qSE3 promoted the absorption of K+ and Na+, induced abscisic acid biosynthesis and abscisic acid signaling pathway gene expression, inhibited the accumulation of reactive oxygen species in seeds, and thus improved the salt tolerance of seeds during germination (He et al., 2019). NaCl promoted the expression of *OsNAC45* in roots, and the knockout of *OsNAC45* resulted in greater ROS accumulations in roots and increased the sensitivity of rice to salt stress (Zhang et al., 2020). Therefore, to obtain more causal genes, we must use more germplasm resources, identify more QTLs, and screen more candidate genes to better understand the genetic mechanism of STG.

Salt tolerance is a complex quantitative trait that is often controlled by multiple genes and the environment. To better understand the genetic mechanism of salt tolerance in rice at the germination stage, many QTLs have been identified by using traditional QTL mapping methods in biparental populations. A total of 16 QTLs were detected during the germination stage under 100 mM NaCl conditions from a RILs that were derived from a cross between Jiucaiqing (salt-tolerant) and IR26 (salt-susceptible; Wang et al., 2011); a total of 11 QTLs were identified for their salt tolerance (1.5% NaCl) at the germination stage by using linkage mapping from a BC_2_F_2:3_ population including 190 accessions that were derived from Dongnong 425 and Changbai 10 (Zheng et al., 2014); 17 QTLs that were related to germination traits under salt stress (80 mM NaCl) in an F2:4 population were detected, which were derived from a cross between the salt-tolerant variety “Gharib” and salt-sensitive variety “Sepidroud” (Mardani et al., 2014); 10 QTLs for five salt stress (0.01 mol L^−1^)-related traits were detected a population of backcross inbred lines, which was derived from a cross between Changhui 891 and 02428 with 3,057 bin markers (Luo et al., 2020); 13 QTLs were identified under H_2_O conditions and salt conditions (300 mM NaCl) by using a BC1F2 population that derived from a WJZ/Nip cross (Zeng et al., 2021); 23 QTLs for germination parameters were detected under 18 dS m^−1^ NaCl solution conditions by using a BIL population that was derived from a backcross of an African rice, ACC9, as the donor and *indica* cultivar Zhenshan97 (ZS97) as the recurrent parent (Nakhla et al., 2021). Biparental populations, although, have played an important role in the past to provide a resource for researchers to work with; construction of populations entails major investments in time, which has therefore limited the number of genes identified to date (Bian et al., 2010).

With the rapid development of NGS (next-generation sequencing) and the existence of high-density SNP markers obtained through resequencing, an increasing number of studies have used GWAS as an efficient strategy to replace the traditional QTL mapping method (Huang et al., 2012). In rice, some studies have applied this method to study rice STG indices. A total of nine significant markers were identified under salt conditions by using 276 *indica* accessions (Cheng et al., 2015); 11 QTLs were identified based on the stress-susceptibility indices (SSIs) of the vigor index (VI) and mean germination time (MGT) in 478 diverse rice accessions (Shi et al., 2017); six quantitative trait nucleotides (QTNs) that affect salt tolerance at the germination stage were identified by GWAS using a panel of 208 rice mini-core accessions (Naveed et al., 2018); 371 QTNs were identified by using six multilocus GWAS methods for the salt tolerance traits of 478 rice accessions at the seed germination stage (Cui et al., 2018); a total of 12 associated peaks were detected by using GWAS in 295 rice accessions during the germination stage (Yu et al., 2018); and 21 QTLs associated with salinity stress were detected in 498 highly diverse rice accessions (Islam et al., 2022). However, to our knowledge, there are still few studies on the identification of QTLs/genes, particularly for the salt tolerance in the germination stage using GWAS in rice. Here, we applied GWAS mapping and used 36,727 SNPs that covered all 12 rice chromosomes in a natural population that consisted of 211 rice accessions to identify QTLs/candidate genes that may contribute to salt tolerance during the rice germination stage with the aim of guiding the breeding of salt-tolerant rice varieties.

## Materials and Methods

### Plant Materials

All materials used in this study were derived from Li et al. (2021). A set of 211 accessions of diverse germplasm accessions representing the major rice-growing regions in China was selected from the International Rice Research Institute (https://www.irri.org/) to comprise the materials used in this work. All data of 36,727 SNPs are published at https://snp-seek.irri.org/. This pool of germplasm accessions consists of two subpopulations, including 130 *indica* and 81 *japonica*, and these 211 rice accessions were divided into two subgroups by using principal component analysis and relationship analysis, which suggested that these germplasm resources had abundant genetic diversity, which was beneficial for performing GWAS.

### Evaluation of Salt Tolerance Germinability

All of the yellow, ripe seeds from each accession were dried at 45°C for 2 days to break seed dormancy. The seeds were surface-sterilized with a 15% sodium hypochlorite solution for 15 min and then rinsed three times with sterile distilled water before the germination experiment. A total of 30 sterilized seeds from each accession were placed on two filter papers soaked with 10 ml of sodium chloride in Petri plates (9 cm) during the germination stage to screen the salinity tolerances, and the concentrations of the sodium chloride solutions were 60 and 100 mM. In the control treatment, the same number of seeds per line was placed on filter papers in Petri dishes that were soaked in 10 ml of distilled water. All Petri dishes were incubated under controlled conditions in a growth chamber at a temperature of 28°C with 12 h each of light and dark. The seed germination time was 7 days, and the numbers of seeds from each accession that germinated were recorded each day. On the 7th day, 10 seedlings from each accession were selected for calculations of shoot length (SL) and root length (RL), and the sodium chloride solutions in the Petri dishes were changed every 2 days. All experiments were repeated three times. The relative germination energy (RGE), relative germination index (RGI), relative vigor index (RVI), relative mean germination time (RMGT), relative shoot length (RSL), and relative root length (RRL) were calculated and subjected to a GWAS, and the calculation formula refers to Yu et al. (2018).

GE = Number of germinated seeds at 4 days/Total number of seeds tested × 100%.
GI = Σ(*G*t/t), where *G*t is the number of seeds that germinated on day t.
VI = GI × SL

MGT = Mean germination time (MGT) = ∑D*n*/∑*n*, where *n* is the number of seeds that germinated on D day, and D is the number of days counted.

Relative value = Value under salt stress/control.

All phenotypic data are presented in Supplementary Table 1. Each index at the two concentrations is divided into five parts. The larger the RMGT value is, the lower the score, and the larger the values of other indices are, the higher the score. The total score of the 12 indices for each accession represents the STG of this accession, and the specific evaluation criteria are shown in Supplementary Table 2.

### Genome-Wide Association Analysis

The Tassel 5.2.73 software and a mixed linear model (MLM) with a PCA matrix (the first five PCs were used) and kinship (K matrix) were used to determine the associations among SNP markers and the 12 phenotypic traits. To ensure data accuracy, the phenotypic data were standardized, and the SNP data (36,727 SNPs) were filtered (in Tassel 5.2.73, the genotypic data were first numerically analyzed, then SNPs with minor allele frequencies <0.05 were removed, and 33,777 SNPs were finally obtained and used for GWAS) before association mapping. Manhattan plots were generated using the Cloud platform (http://www.cloud.biomicroclass.com/).

### Candidate Gene Analyses

The LD blocks were used to identify candidate gene regions using the Haploview 4.2 software (Barrett et al., 2005). The SNPs with the most significant associations in a block were identified as the leading SNPs, and LD blocks containing significantly associated SNPs were defined as candidate genomic regions. Differentially expressed genes were identified using the Plant Public RNA-seq Database (Yu et al., 2022). The expression patterns of the differentially expressed genes were analyzed using publicly available microarray data (http://www.genevestigator.com/). Haplotype analyses were performed using RiceVarMap V2.0 (Zhao et al., 2015). Information on candidate genes was collected and classified by the NCBI (https://www.ncbi.nlm.nih.gov/), China Rice Data Center (https://www.ricedata.cn/), and the Rice Genome Annotation Project (http://rice.uga.edu/index.shtml).

### Quantitative Real-Time PCR Analysis

Embryos of seeds that were germinated in water and in 60 mM NaCl solutions for 2 days were collected for expression analysis. Total RNA was prepared using a MiniBEST Plant RNA Extraction kit (Takara, China). The corresponding sequences of these genes were obtained from the Rice Genome Annotation Project (http://rice.uga.edu/index.shtml). The primers (e.g., *LOC_Os03g13560*-F: CAGATTGTGATATGGTGTTCGGC; *LOC_Os03g13560*-R: GGAGACTGAGAAGCTGTCATCAT; *LOC_Os03g13840*-F: CTGGACAAGGTACTGGAGGAGTA; *LOC_Os03g13840*-R: CGTTCTTTCCAGACACCTCTACA; *LOC_Os03g14180*-F: AGGTGAGGATGCGGTTCG; and *LOC_Os03g14180*-R: CGCTCACAGGCTCACATCC) were designed based on the CDSs of the corresponding genes by using Primer3Plus (https://www.primer3plus.com/). OsActin was used as the internal control. Real-time PCR was carried out using the SYBR Green method, and the relative expression levels were calculated using the 2^−ΔΔCT^ method.

### Statistical Analysis and Mapping

The mean values and standard errors of the phenotypic data were calculated using Microsoft Excel 2010, and the correlation coefficients were calculated using the SPSS 26.0 software. Box plots were created using the OriginPro 2021 software.

## Results

### Phenotypic Analysis of the 211 Rice Accessions and Correlations Among STG Indices

**Figure 1:**
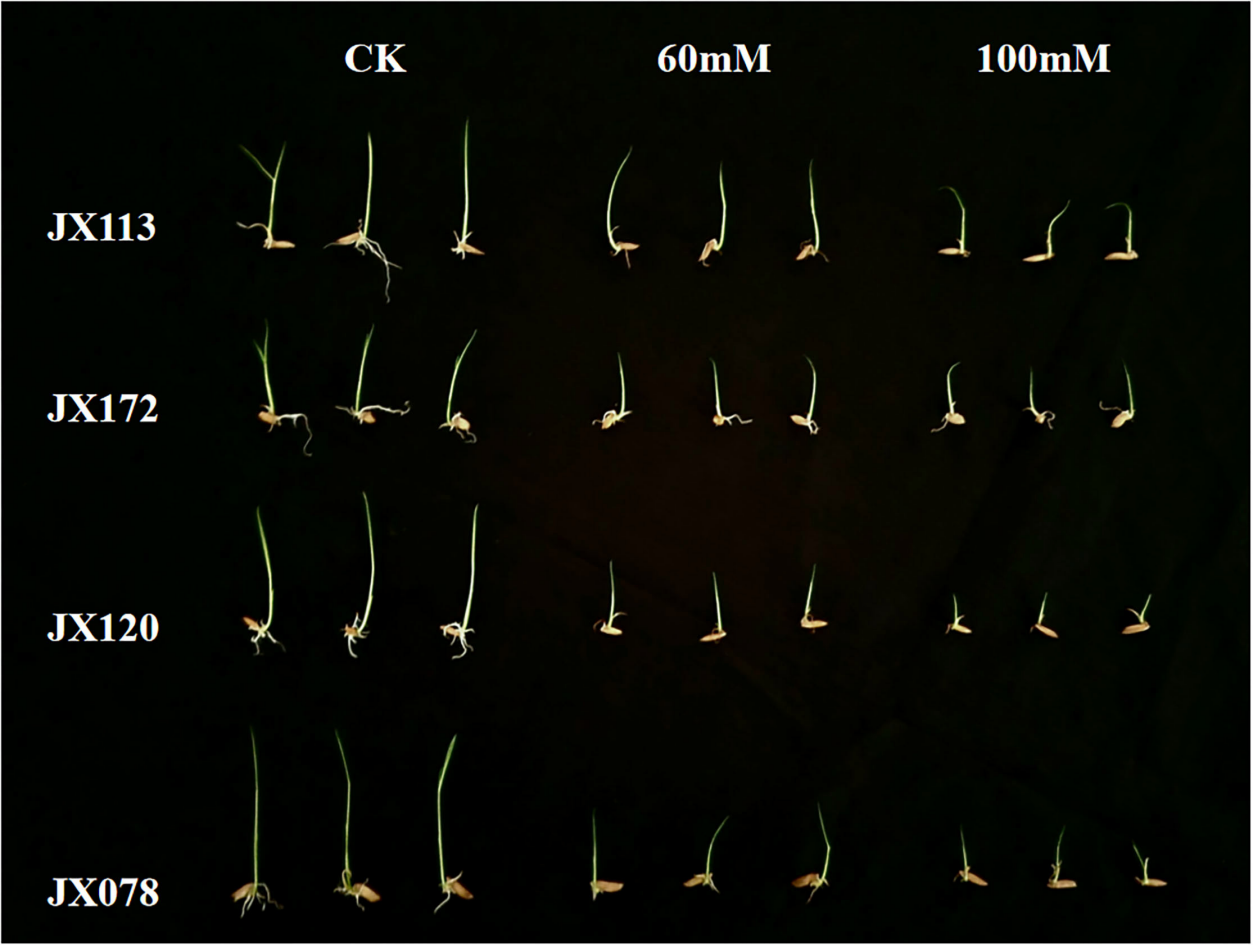
Comparison of growth status of two salt-tolerant rice varieties (JX 113 and JX 172) and two salt-sensitive rice varieties (JX 120 and JX 078) after 7 days of control treatment and 60 and 100 mM NaCl solution.

In this study, two sodium chloride concentrations (e.g., 60 and 100 mM) were selected to conduct an STG assessment. After 7 days of germination, the growth states of 211 rice varieties under the two salt solutions were inhibited compared with the control treatment, but the distribution of the inhibition degree among different varieties was different (Figure 1), which indicates that both sodium chloride concentrations are suitable for screening salt-tolerant and salt-sensitive varieties. The salt-tolerant rice varieties identified in this study can be used as excellent parent resources in subsequent breeding projects.

**Figure 2:**
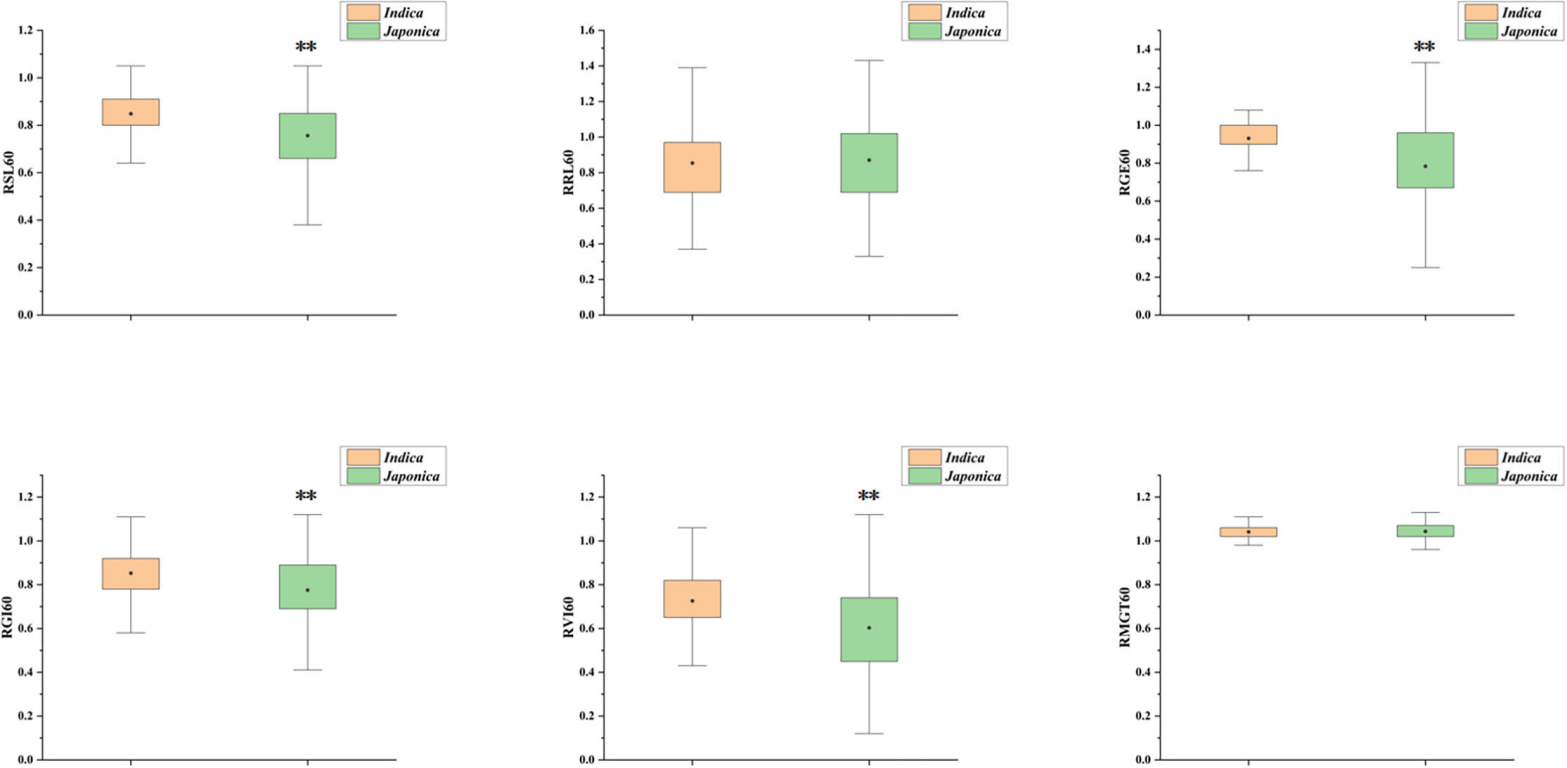
Box plot for comparison of six indices between *indica* and *japonica* subgroups under 60 mM NaCl. The yellow box represents *indica*, the green box represents *japonica*, the black dot in the box represents the median, and the value range of the box is 25–75%, **Indicates significance at the 1% level.

**Figure 3:**
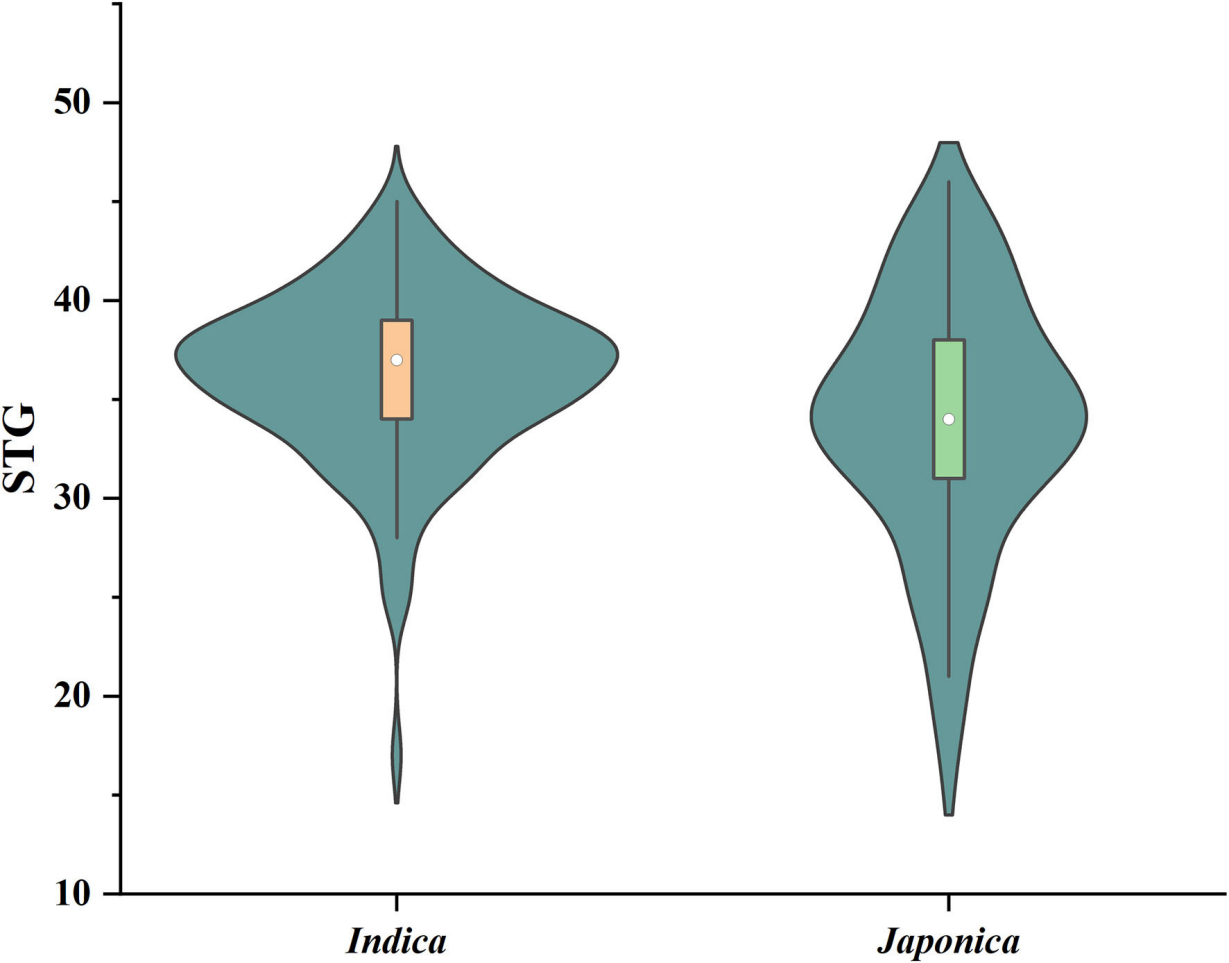
Violin diagram of the distribution of STG values of *indica* and *japonica* subgroups. Boxes in violins range in value from 25 to 75%, with white dots representing the median.

Six indices obtained under two sodium chloride concentrations were used to evaluate the STG in this natural population, and all of the indices showed large phenotypic variations among the rice accessions (Table 1). In particular, except for RMGT60 and RMGT100, the values of all indices were widely distributed, and the coefficients of variation were very large. This result suggested that these STG indices may be suitable for performing a GWAS. In addition, six STG-related indices were compared among different subgroups in the population (Figure 2; Supplementary Figure 1). Under the 60 mM NaCl condition, the RSL, RGE, RGI, and RVI values of the *japonica* group were significantly lower than those of the *indica* group (*p* < 0.01), but the RRL and RMGT values exhibited no significant differences between the two subgroups. On contrary, at an NaCl concentration of 100 mM, the RSL, RGE, RGI, and RVI values in the *indica* subgroup were higher than those in the *japonica* subgroup, but the RRL values in the *japonica* subgroup were higher than those in the *indica* subgroup, and there were no significant differences in the RMGT values between the two subgroups. We used these six indices to define a scoring standard to screen varieties with high STG, and the results showed that most *indica* rice varieties generally had greater STG than the *japonica* rice varieties (Figure 3).

**Table 1:** The distribution of STG indices in 211 rice accessions.

**Indices**	**Range (%)**	**Mean (%)**	**SD**	**Coefficient of**
				**variation (%)**
RSL60	35–161	81	0.15	18.34
RRL60	1–176	86	0.25	28.52
RGE60	8–167	87	0.21	23.94
RGI60	26–137	82	0.14	17.44
RVI60	9–130	68	0.19	27.39
RMGT60	95–118	104	0.03	3.31
RSL100	21–112	64	0.15	23.10
RRL100	1–130	60	0.20	33.89
RGE100	1–112	73	0.26	36.11
RGI100	20–112	68	0.17	24.40
RVI100	5–98	45	0.17	37.48
RMGT100	98–123	108	0.05	4.28

**Figure 4:**
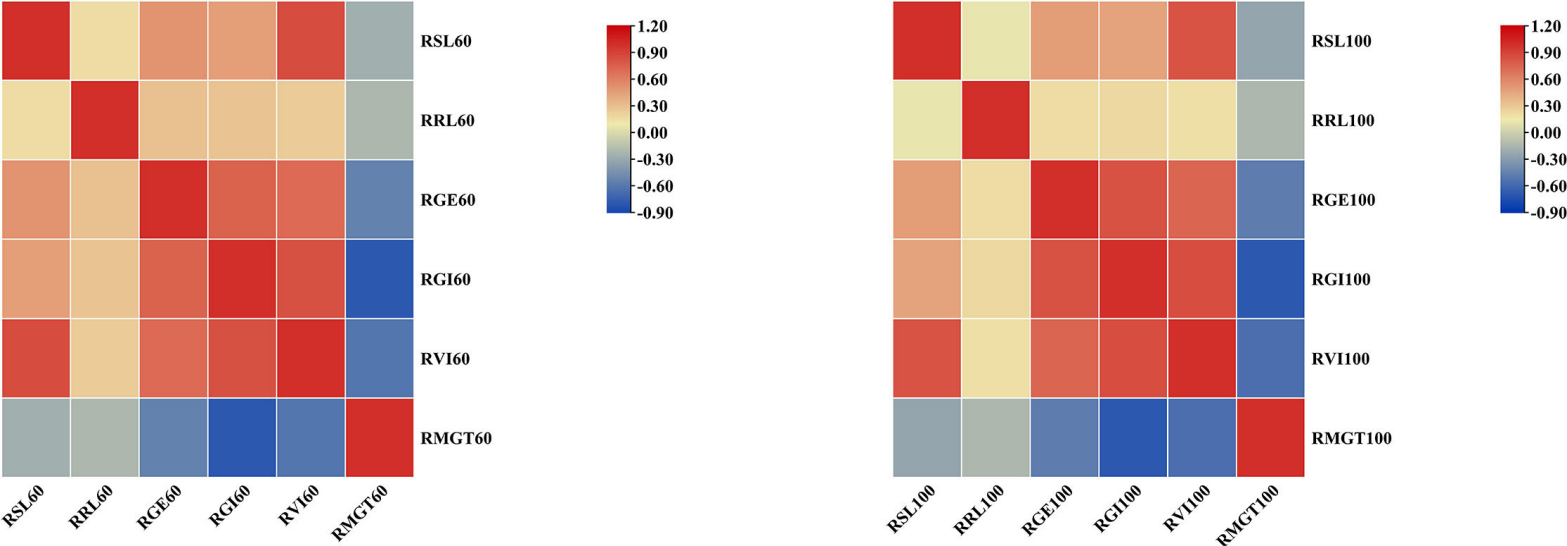
Heat map of correlation analysis between six indices under two different concentrations of salt solution. Blocks that tend to be red or blue indicate greater absolute values of the correlation coefficients.

To determine how the mean values of the different STG indices for each accession compared to each other, pairwise Pearson's correlation analysis was conducted (Figure 4). Under the 60 mM NaCl condition, the RSL, RRL, RGE, RGI, and RVI values were all positively correlated to varying degrees, and they were all significantly negatively correlated with the RMGT. However, the RSL had no correlation with RRL at a concentration of 100 mM, but both were significantly positively correlated with the RGE, RGI, and RVI values, and the RMGT was significantly negatively correlated with the other five indices. These results suggest that the RGE, RGI, RVI, and RMGT indices might share genetic pathways under both the 60 and 100 mM salt treatments. In addition, a correlation analysis between the two treatments was conducted. The results showed that there were significant positive correlations between the two, and the correlation coefficient was 0.568.

### Identification of QTLs for Salt Tolerance Germinability by GWAS

**Figure 5:**
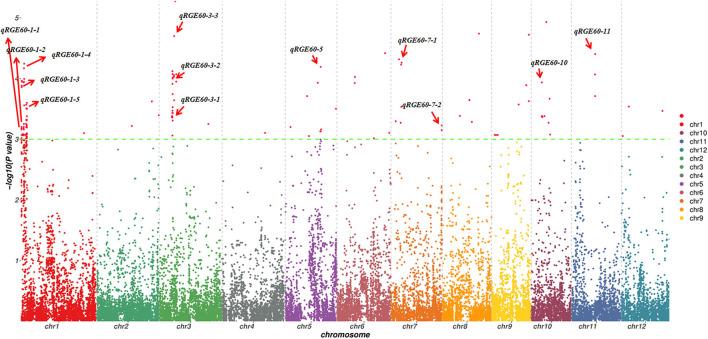
Manhattan plots of GWAS for RGE under 60 mM NaCl. The red arrow indicates QTLs detected from six indices.

To investigate the possible genetic architecture of STG, a GWAS was conducted, and Manhattan plots were generated to illustrate the significant SNP associations with STG (Figure 5; Supplementary Figure 2). A total of 43 QTLs, including 15 for RGE, 6 for RGI, 7 for RVI, 3 for RMGT, 1 for RSL, and 11 for RRL, which were associated with STG, were identified in rice (Table 2). On chromosome 1, 10 QTLs were discovered for the first time, among which *qRGE60-1-2* overlapped with *qRGI60-1-1* and was located at 1.2 Mb, and *qRVI60-1-2* and *qRGI60-1-4* were co-localized at 3.5 Mb. In addition, nine QTLs were co-localized with those described in previous studies: *qRRL-100-1* was located at 0.2 Mb and overlapped with *OsCOIN* (Liu et al., 2007); *qRGE60-1-2, qRGI60-1-2* and *qRMGT60-1-1* overlapped with each other, and a previously cloned gene related to salt stress, *OsHsp17.0* (Zou et al., 2012), was located in this region; at ~3 Mb, three QTLs (e.g., *qRGI60-1-3, qRVI60-1-1*, and *qRGE60-1-4*) were co-located with each other, and *OsDREB2A* (Mallikarjuna et al., 2011) was located in this region; and two QTLs related to RRL, namely, *qRRL60-1-2* and *qRRL60-1-3*, overlapped with *OsPLD*α*1* (Shen et al., 2011) and *OrbHLH001* (Chen et al., 2013), respectively. These QTLs had explained phenotypic variances (*R*^2^) ranging from 5.48% for *qRGE60-1-2* to 9.76% for *qRGE60-1-3*. Only three QTLs were identified on chromosome 2, among which *qRRL60-2-1* overlapped with a previously reported STG QTL, *qGR-3d2* (Nakhla et al., 2021), and the peak SNP was chrA02_4380604. Seven QTLs mapped to chromosome 3 and *qRGE60-3-1* and *qRVI60-3-1* shared the same SNP peak at 7.66 Mb, three QTLs from two indices (e.g., *qRGE60-3-3, qRGE100-3*, and *qRVI60-3-2*) shared the same SNP peak at 9.85 Mb, while this QTL region contains *OsMAPK5* (Xiong and Yang, 2003), a salt stress-related gene. These QTLs had explained phenotypic variances (R^2^) ranging from 7.46% for *qRVI60-3-1* to 12.27% for *qRGE60-3-3*. In the remaining chromosomes, 5, 6, 7, 9, 10, and 11, five QTL regions were co-localized with previously reported salt-tolerant QTLs or genes (Park et al., 2010; Li et al., 2015; Nakhla et al., 2021), and the explained phenotypic variances (*R*^2^) ranged from 5.41% for *qRVI60-5* to 10.14% for *qRGE60-11*. We noted that many RGE, RGI, RVI, and RMGT QTLs mapped to identical locations with the same peak SNPs. This supports our previous speculation that these four indices might share genetic pathways.

**Table 2:** Summary of the significant SNPs detected by GWAS and the overlapping QTLs reported previously.

**QTL ID**	**Trait**	**Chr**.	**Peak SNPs**	***P*** **-value**	***R*** ^**2**^	**Previous QTL/genes**	**References**
*qRRL-100-1*	RRL	1	201819	7.85E-04	0.070961901	*OsCOIN*	(Liu et al., 2007)
*qRGE60-1-1*	RGE	1	455562	6.31E-04	0.073714432		
*qRGE60-1-2*	RGE	1	1243681	8.25E-04	0.054838078		
*qRGI60-1-1*	RGI	1	1243681	1.97E-04	0.089011191		
*qRGE60-1-3*	RGE	1	1957926	5.70E-05	0.097587946	*OsHsp17.0*	(Zou et al., 2012)
*qRGI60-1-2*	RGI	1	1957926	4.53E-04	0.076102635	*OsHsp17.0*	(Zou et al., 2012)
*qRMGT60-1-1*	RMGT	1	1957926	7.99E-05	0.094079583	*OsHsp17.0*	(Zou et al., 2012)
*qRGI60-1-3*	RGI	1	3046380	6.16E-04	0.072982743	*OsDREB2A*	(Mallikarjuna et al., 2011)
*qRVI60-1-1*	RVI	1	3046380	7.17E-04	0.071413901	*OsDREB2A*	(Mallikarjuna et al., 2011)
*qRGE60-1-4*	RGE	1	3046380	2.70E-04	0.08148945	*OsDREB2A*	(Mallikarjuna et al., 2011)
*qRMGT60-1-2*	RMGT	1	3478635	3.18E-04	0.07974077		
*qRVI60-1-2*	RVI	1	3493294	3.16E-04	0.079821455		
*qRGI60-1-4*	RGI	1	3493294	4.43E-04	0.076340862		
*qRGE60-1-5*	RGE	1	3511964	6.34E-04	0.082964523		
*qRRL60-1-1*	RRL	1	3545015	2.15E-04	0.086175194		
*qRMGT60-1-2*	RMGT	1	3478635	3.18E-04	0.07974077		
*qRRL60-1-2*	RRL	1	3939680	2.16E-04	0.086169671	*OsPLDα1*	(Shen et al., 2011)
*qRGI60-1-5*	RGI	1	15977602	3.83E-04	0.077840812		
*qRRL60-1-3*	RRL	1	42421352	1.45E-04	0.090155653	*OrbHLH001*	(Chen et al., 2013)
*qRRL60-2-1*	RRL	2	4380604	6.69E-04	0.075583000	*qGR-3d2*	(Nakhla et al., 2021)
*qRRL60-2-2*	RRL	2	18813593	5.33E-04	0.077148185		
*qRSL100-2*	RSL	2	19661213	1.88E-04	0.086493361		
*qRGE60-3-1*	RGE	3	7668652	7.57E-05	0.094665597		
*qRVI60-3-1*	RVI	3	7668652	5.28E-04	0.074581176		
*qRGE60-3-2*	RGE	3	8660029	2.00E-05	0.108593017		
*qRGE60-3-3*	RGE	3	9852079	5.32E-06	0.122680821	*OsMAPK5*	(Xiong and Yang, 2003)
*qRGE100-3*	RGE	3	9852079	4.98E-04	0.075150154	*OsMAPK5*	(Xiong and Yang, 2003)
*qRVI60-3-2*	RVI	3	9852079	2.83E-04	0.080963707	*OsMAPK5*	(Xiong and Yang, 2003)
*qRMGT100-3*	RMGT	3	33227077	9.80E-04	0.085821366		
*qRVI60-5*	RVI	5	14178828	8.95E-04	0.054078023		
*qRGE60-5*	RGE	5	20392181	6.45E-05	0.079537025		
*qRRL60-6*	RRL	6	5117960	2.28E-04	0.085628982	*qGR-7d6*	(Nakhla et al., 2021)
*qRGE60-7-1*	RGE	7	5888791	5.92E-05	0.097180181		
*qRGE60-7-2*	RGE	7	29343026	5.93E-04	0.077278782		
*qRRL60-9*	RRL	9	16654472	1.73E-04	0.088530434	*OsDSG1*	(Park et al., 2010)
*qRGI60-9*	RGI	9	17191317	9.93E-05	0.100093083	*OsbHLH120*	(Li et al., 2015)
*qRVI60-9*	RVI	9	17191317	3.30E-04	0.084884769	*OsbHLH120*	(Li et al., 2015)
*qRVI100-9*	RVI	9	17191317	8.91E-05	0.095534518	*OsbHLH120*	(Li et al., 2015)
*qRRL60-10-1*	RRL	10	4285687	7.40E-05	0.097070419		
*qRRL100-10*	RRL	10	4665777	3.95E-04	0.079672322		
*qRGE60-10*	RGE	10	6107128	1.16E-04	0.091425897		
*qRRL60-10-2*	RRL	10	7248029	2.45E-04	0.084358531		
*qRGE60-11*	RGE	11	13756114	3.96E-05	0.101443433		
*qRGE100-11*	RGE	11	19317926	3.80E-04	0.079063947		

### Candidate Gene Prediction and Expression Profiling

**Figure 6:**
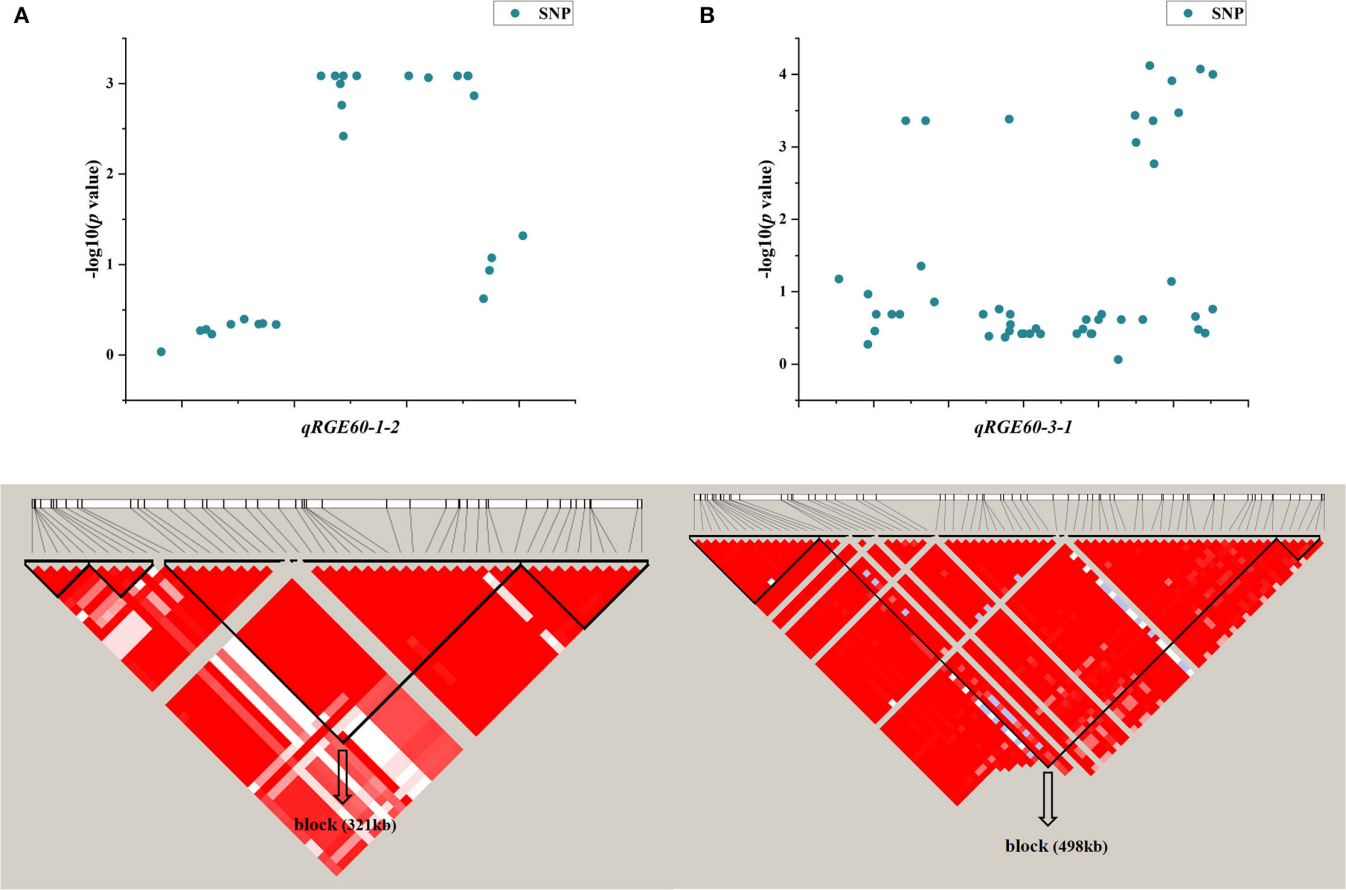
LD block of candidate region estimation of two major QTLs, namely, *qRGE60-1-2* **(A)** and *qRGE60-3-1* **(B)**.

One QTL, *qRGE60-1-2* (*qRGI60-1-1*), which controls both RGE and RGI on chromosome 1, was identified by GWAS. According to the LD decay analysis, a 321-kb region was identified as the candidate region for *qRGE60-1-2* (Figure 6A), which contained 52 genes, including 25 functionally annotated genes, 18 expressed proteins with unknown functions, four retrotransposon proteins, three transposon proteins, and two hypothetical proteins (Supplementary Table 3). To decrease the number of candidate genes, genes categorized as expressed proteins, hypothetical proteins, retrotransposons, and transposons were discarded. Among the 25 functionally annotated genes, nine genes were found to exhibit significant differences in their expression levels between the control and salt treatments according to the Plant Public RNA-seq Database (|logFoldChange| ≥ 1.5), among which seven genes were upregulated under salt stress, one gene was downregulated, and the other gene had different expression trends under the different projects (Supplementary Table 4).

Another QTL, *qRGE60-3-1* (*qRVI60-3-1*), is also considered a candidate, which controls both RGE and RVI on chromosome 3. According to the LD decay analysis, a 489-kb region was identified as the candidate region (Figure 6B), and it contained 42 functionally annotated genes (Supplementary Table 3). According to the RNA-seq database (|logFoldChange| ≥ 1.5), the expression levels of seven genes were changed under salt stress, among which four genes were upregulated and three genes were downregulated (Supplementary Table 4).

**Figure 7:**
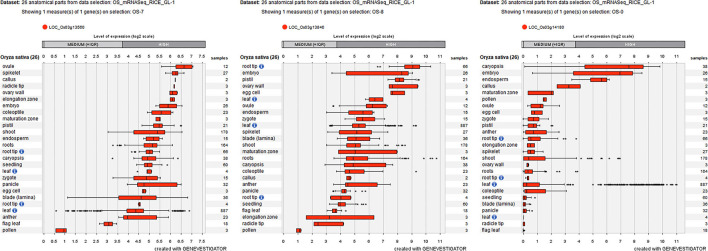
Three differentially expressed genes (*LOC_Os03g13560, LOC_Os03g13840*, and *LOC_Os03g14180*) are highly expressed in embryos labeled by Geneinvestigator. The * represent discrete point.

To further decrease the number of candidate genes, the expression patterns of these 16 differentially expressed genes were identified by using Genevestigator. Fortunately, nine genes (e.g., *LOC_Os01g02930, LOC_Os01g02940, LOC_Os01g03330, LOC_Os01g03340, LOC_Os01g03360, LOC_Os03g13560, LOC_Os03g13840, LOC_Os03g14050*, and *LOC_Os03g14180*) were determined to be highly expressed in embryos (Figure 7; Supplementary Figure 3), which suggested that these nine genes are involved in rice seed germination under salt stress and could be candidates for STG.

### Natural Allelic Variations of Candidate Genes Contribute to Salt Tolerance Germinability

**Figure 8:**
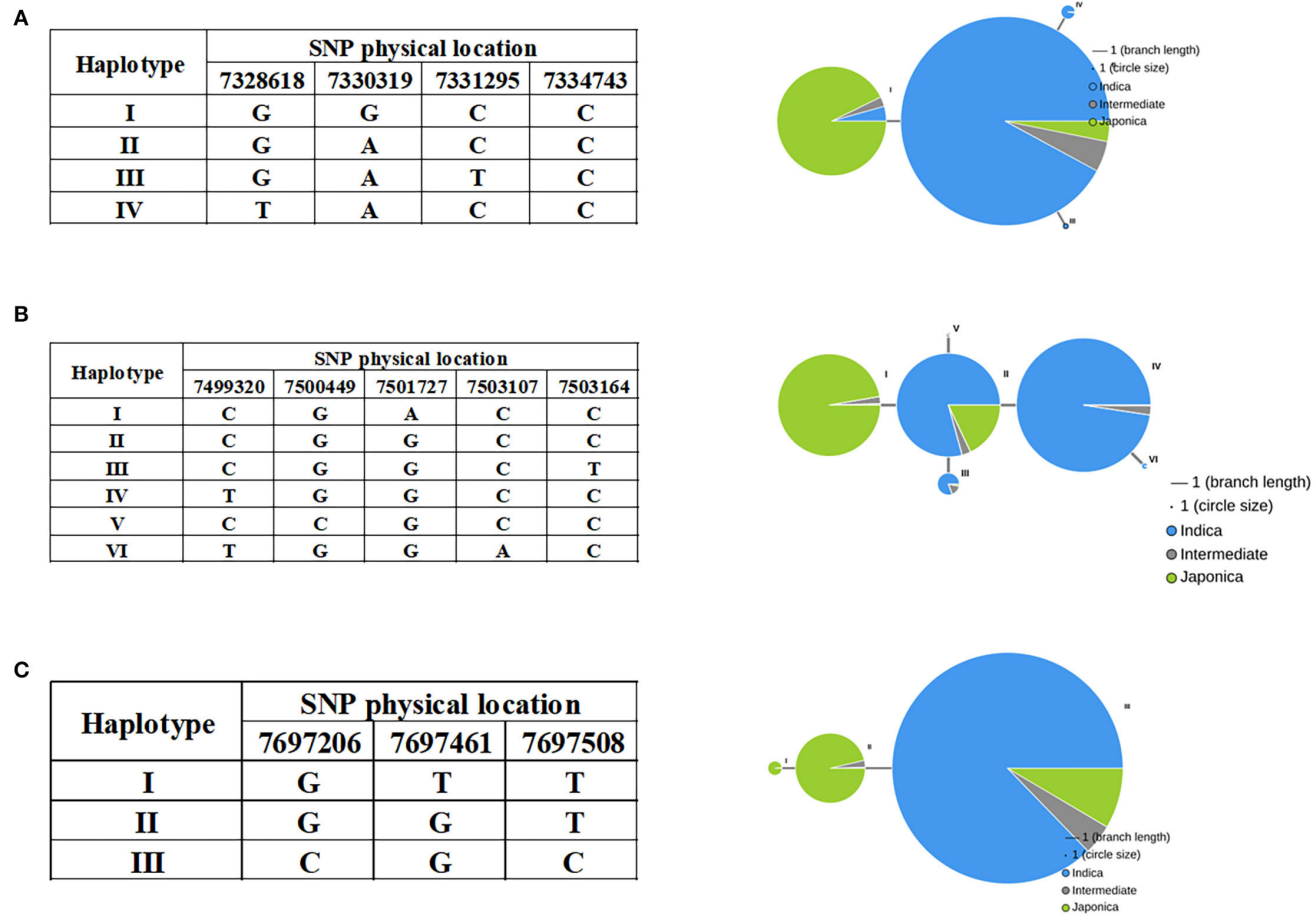
Haplotypes of *LOC_Os03g13560* **(A)**, *LOC_Os03g13840* **(B)**, and *LOC_Os03g14180* **(C)** are associated with STG in rice.

As noted above, most *indica* rice varieties in our population had higher STG levels than *japonica* rice varieties, and we therefore investigated whether variations in the candidate gene alleles contributed to these differences. Haplotype analyses were performed using all non-synonymous SNPs within their ORFs by RiceVarMap v2.0. Finally, it was found that three genes had different haplotypes between *indica* and *japonica*. The *LOC_Os03g13560* gene harbored a total of four non-synonymous SNPs, namely, vg0307328618 (G/T), vg0307330319 (A/G), vg0307331295 (C/T), and vg0307334743 (C/T). Four distinct haplotypes, including two major haplotypes, namely, Hap I and Hap II, were identified based on the four non-synonymous SNPs in cultivated rice and exhibited large genetic differences between *indica* and *japonica* (Figure 8A). There are five non-synonymous SNPs in *LOC_Os03g13840*, namely, vg0307499320 (C/T), vg0307500449 (G/C), vg0307501727 (G/A), vg0307503107 (C/A), and vg0307503164 (C/T). Six major haplotypes were observed at *LOC_Os03g13840*, which encodes a senescence-associated protein. Hap I was present in *japonica*, and Hap II, III, and IV were present primarily in *indica* (Figure 8B). Interestingly, Hap II was carried in ~16% of *japonica* accessions, which indicated that elite *indica* alleles had been introgressed into *japonica* accessions through breeding to the extent that a small percentage of the *japonica* accessions (such as JX172 and JX224) included in our population had high STG levels. A total of three non-synonymous SNPs [e.g., vg0307697206 (C/G), vg0307697461 (G/T), and vg0307697508 (C/T)] in *LOC_Os03g14180* identified three haplotypes (Figure 8C). The vast majority of *indica* accessions were Hap III, while the *japonica* accessions were Hap I or Hap II. Taken together, these results indicate that the natural allelic variations in these three candidate genes appeared to be associated with the different STG levels observed among the 211 rice accessions.

**Figure 9:**
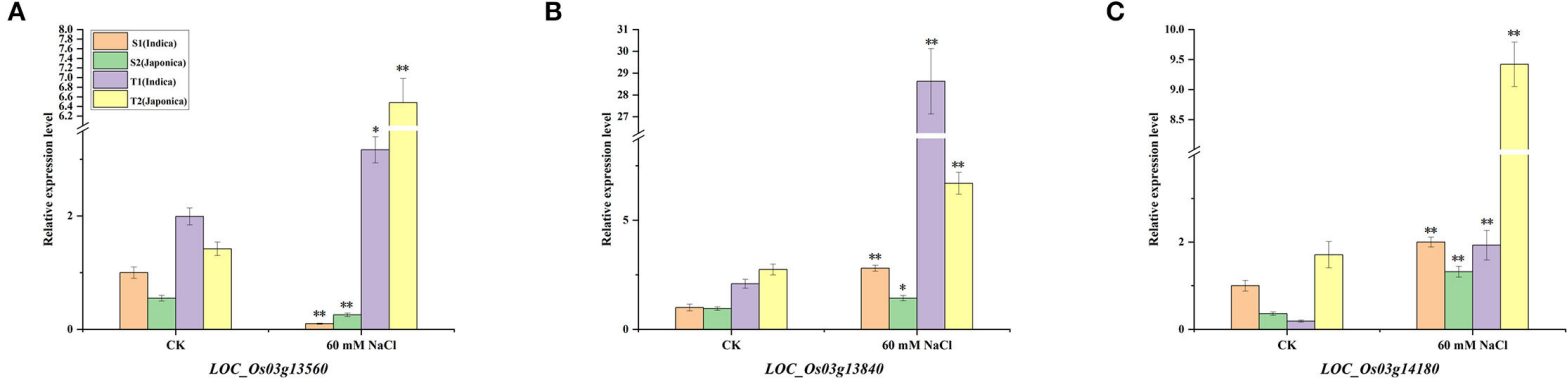
Expression patterns of *LOC_Os03g13560* **(A)**, *LOC_Os03g13840* **(B)**, and *LOC_Os03g14180* **(C)** under normal growth conditions and salt stress conditions. *Indicates significance at the 5% level, **Indicates significance at the 1% level.

To further verify whether the three genes respond to salt stress and have *indica*–*japonica* specificity in the individuals examined in our population, quantitative real-time PCR (qRT-PCR) was performed with two salt-sensitive varieties (e.g., S1 and S2) and two salt-tolerant varieties (e.g., T1 and T2) under control and 60 mM NaCl conditions. The results showed that these three genes were differentially expressed under salt stress (Figure 9). For *LOC_Os03g13560*, the expressions in salt-sensitive cultivars were downregulated, while the expressions in salt-tolerant cultivars were upregulated under salt stress (Figure 9A). In addition, the downregulation times of S1 (*indica*) were much higher than those of S2 (*japonica*) in the two salt-sensitive varieties, and the upregulation times of T2 (*japonica*) were much higher than those of T1 (*indica*) in the two salt-tolerant varieties. The *LOC_Os03g13840* gene was upregulated under salt stress in both salt-sensitive and salt-tolerant varieties, and the upregulated *LOC_Os03g13840* expressions in the two *indica* rice varieties were higher than those in *japonica* rice (Figure 9B). The *LOC_Os03g14180* gene responded to salt stress in all four rice varieties and was upregulated under 60 mM NaCl conditions (Figure 9C). Similarly, the changes in the expression levels of *LOC_Os03g14180* were *indica*–*japonica* specific. After salt stress, the change times of S2 were higher than those of S1, and the change times of T1 were higher than those of T2.

## Discussion

Direct seeding of rice has become popular in many Asian countries because of its time-saving, labor-saving, and cost-effective advantages. However, to develop high-yield and high-quality rice varieties for use in the direct seeding mode, rice seeds must have strong germination abilities under various abiotic stresses and strong growth abilities after abiotic stresses. Therefore, cultivating rice varieties with high STG has become an inevitable trend. In our study, six indices containing four indices (e.g., RGE, RGI, RVI, and RMGT) associated with seed germination and two indices (e.g., RSL and RRL) associated with seed growth capacity after salt stress were used to evaluate STG levels. These indices have been used successfully in previous GWAS studies on salt tolerance at the rice germination stage (Shi et al., 2017; Yu et al., 2018). Our study showed that additions of salt solutions inhibited seed germination compared with the control treatment; that is, the GE, GI, VI, and MGT values of seeds decreased, and the inhibition degrees also increased with increasing salt solution concentrations (Table 1). Despite salt stress, nearly all rice accessions germinated successfully in the following days, but this does not mean that salt stress prolonged only the germination time and did not have other effects because after 7 days of salt stress, we found that the SL and RL values of rice were reduced compared to the control group, which suggested that salinity can influence the germination quality of seeds.

Different conclusions have been reached in different studies on the salt tolerance of different rice subgroups in the germination stage. According to Shi et al. (2017), *japonica* varieties had higher tolerance to salt stress at the germination stage than did the other subgroups, which was determined by measuring the SSIs of VI and GR at 10 days. However, Yu et al. (2018) and Islam et al. (2022) suggested that the salt tolerance levels during the seed germination stage are not well-correlated with rice subgroups. In our study, a scoring rule was developed based on six indices under two different salt solution concentrations to determine the STG level of each rice accession, and the results showed that the STG of *indica* rice was generally significantly higher than that of *japonica* rice. No significant differences in RMGT values were observed between the two subgroups at either salt solution concentration, but the RSL, RGE, RGI, and RVI values of *indica* for the two salt solution concentrations were significantly greater than those of *japonica*. There were no significant differences between the two subgroups under the 60 mM condition, but the RRL values of *japonica* were greater than those of *indica* under the 100 mM condition, which was probably because with the increased salt solution concentration, salt-sensitive rice varieties needed to extend their roots longer to increase their water uptake areas to survive.

The purpose of using a variety of indices to evaluate rice STG is to obtain a more comprehensive understanding of the genetic mechanisms of rice varieties subjected to salt stress in the germination stage. The correlations among the six indices were analyzed, and the results showed that the RGE, RGI, and RVI values were significantly positively correlated with each other and were negatively correlated with RMGT regardless of whether concentrations of 60 or 100 mM were used, which may indicate that the four indices are regulated by a consistent genetic pathway. This inference was confirmed by the subsequent GWAS analysis.

A total of 33 distinct QTL regions were identified by GWAS using six indices and two concentrations, among which 10 QTLs were found to be co-localized in the same or overlapping regions of previously reported salt stress QTLs/genes. The QTL *qRRL-100-1* overlapped with *OsCOIN*, a gene encoding ring zinc finger protein that is located in the nucleus and plasma membrane, and *OsCOIN* is expressed in all organs of rice and is strongly induced by low temperatures, ABA, salt and drought. Overexpression of *OsCOIN* upregulated the expression of *OsP5CS* and increased intracellular proline contents, which ultimately significantly enhanced the resistance of transgenic rice to cold, salt, and drought (Liu et al., 2007). A small heat shock protein, *OsHSP17.0*, appeared in a QTL region that was repeatedly detected (e.g., *qRGE60-1-3*/*qRGI60-1-2*/*qRMGT60-1-1*) in our study. The germination capacities of *OsHsp17.0*-overexpressing transgenic lines in NaCl was stronger than that of wild-type plants, and phenotypic analysis showed that transgenic rice lines were more tolerant to salt stress than WT rice lines (Zou et al., 2012). An AP2/EREBP transcription factor, *OsDREB2A*, was located in a candidate region that contains three co-localized QTLs (e.g., *qRGI60-1-3, qRVI60-1-1*, and *qRGE60-1-4*). *OsDREB2A* expression was induced by high salinity stress (Dubouzet et al., 2003), and its overexpressed lines were significantly tolerant to osmotic stress, drought stress, salt stress, and dehydration stress, and their growth performance was enhanced (Cui et al., 2011; Mallikarjuna et al., 2011). Five RRL-related QTLs (e.g., *qRRL60-1-2, qRRL60-1-3, qRRL60-2-1, qRRL60-6*, and *qRRL60-9*) that were evaluated in our study were co-localized with previously reported salt stress QTLs/genes (e.g., *OsPLD*α*1, OrbHLH001, qGR-3d2, qGR-7d6*, and *OsDSG1*). *OsPLD*α*1* is involved in the salt tolerance of rice by regulating the activity and expression of H+-ATPase (Shen et al., 2011). Overexpression of *OrbHLH001*, a putative helix-loop-helix transcription factor, causes increased expression of AKT1 and maintains ionic balance under salt stress in rice (Chen et al., 2013). *qGR-3d2* and *qGR-7d6* are two QTLs associated with germination rates that were detected in the germination stage (Nakhla et al., 2021). *OsDSG1* is a gene that affects seed germination, and loss of this gene results in delayed seed germination, shorter plants, and greater tolerance to high salt and drought stresses (Park et al., 2010). A QTL containing three co-located QTLs (e.g., *qRGE60-3-3, qRGE100-3*, and *qRVI60-3-2*) on chromosome 3 explained the largest phenotypic variations in our study, and this QTL region contained a characterized salt-tolerant gene, *OsMAPK5*, which is a mitogen-activated protein that can positively regulate the tolerance of rice to drought, salt, and cold stresses (Xiong and Yang, 2003). Similarly, a QTL (e.g., *qRGI60-9*/*qRVI60-9*/*qRVI100-9*) is present in 17.19 Mb of chromosome 9 that explains the large phenotypic variations. This region contains a basic helix-loop-helix transcription factor called *OsbHLH120*, and the expression of *OsbHLH120* in IL392 (an introgression line) roots was higher than that of Yuefu (parent) under PEG and salt stresses (Li et al., 2015). In addition, 23 new loci were identified in this study, among which three loci contained more than one QTL. These new QTLs detected by multiple indices will provide important genetic resources for identifying candidate salt genes.

Hence, the exact candidate intervals were determined by LD blocks for two new QTLs, *qRGE60-1-2* and *qRGE60-3-1*, with high explained phenotypic variations, which were detected in multiple indices. According to a previous study (Neang et al., 2020), only 67 functionally annotated genes were retained in these two QTLs. We used multiple databases to determine the expression patterns of these functional annotation genes and whether they responded to abiotic stress. Finally, nine genes that respond to salt stress and are highly expressed in embryos were selected as candidate genes. Interestingly, the haplotype analysis suggested that the haplotypes of three candidate genes (e.g., *LOC_Os03g13560, LOC_Os03g13840*, and *LOC_Os03g14180*) exhibited some *indica*–*japonica* specificity, which was consistent with our phenotypic results that the STG levels of *indica* and *japonica* rice were significantly different. *LOC_Os03g13560* encodes a hydroxyproline-rich glycoprotein family protein. A previous study revealed that hydroxyproline-rich glycoprotein family proteins may be involved in the chilling response of soybean (Xu et al., 2016). *LOC_Os03g13840* encodes senescence-associated protein (SAP). A gene ontology classification was used to predict its function, and the results showed that there were two major GO accessions for this candidate gene, namely, GO:0009628 (response to abiotic stimulus) and GO:0006950 (response to stress). Ubaidillah et al. (2013) revealed that *OsSAP* was both highly and rapidly expressed in response to drought stress. *LOC_Os03g14180* is a rice heat shock protein gene named *OsHSP26.7*. The transcript levels of *OsHSP26.7* were also high in the imbibed seed embryos but were very low in other organs and were significantly induced by heat treatment and high salt stress (Zou et al., 2009). In conclusion, the identification of candidate genes and their elite haplotypes for QTLs provides important information to identify the causal genes and facilitate further validation and application of the identified QTLs in trait improvements.

## Data Availability Statement

The datasets presented in this study can be found in online repositories. The names of the repository/repositories and accession number(s) can be found at: https://ngdc.cncb.ac.cn/gvm/, GVM000333.

## Author Contributions

CLi designed experiments, analyzed data, and wrote the manuscript. CLi, CLu, and BZ performed all the phenotypic evaluations. MY, GW, PW, QC, YW, QZ, SH, TH, and HH participated in performing experiments. JB conceived and supervised the experiments. All authors have read and agreed to the published version of the manuscript.

## Funding

This study was supported by a grant (32160485) from the National Natural Science Foundation of China, a grant (YC2021-S341) from the Innovation Fund Designated for Graduate Students of Jiangxi Province, and College Students Innovative Entrepreneurial Training Plan Program (Grant No. 202210410002). These funding institutions provided financial support for material collection, high-throughput sequencing, and data analysis in this study.

## Conflict of Interest

The authors declare that the research was conducted in the absence of any commercial or financial relationships that could be construed as a potential conflict of interest.

## Publisher's Note

All claims expressed in this article are solely those of the authors and do not necessarily represent those of their affiliated organizations, or those of the publisher, the editors and the reviewers. Any product that may be evaluated in this article, or claim that may be made by its manufacturer, is not guaranteed or endorsed by the publisher.

## Supplementary Material

Supplementary Figure 1 Box plot for comparison of six indices between *indica* and *japonica* subgroups under 100 mM NaCl. The yellow box represents *indica*, the green box represents *japonica*, the black dot in the box represents the median, and the value range of the box is 25–75%, ^**^Indicates significance at the 1% level.

Supplementary Figure 2 Manhattan plots of GWAS for RGI **(A)**, RVI **(B)**, RMGT **(C)**, RRL **(D)**, under 60 mM NaCl and RGE **(E)**, RVI **(F)**, RMGT **(G)**, RSL **(H)**, and RRL **(I)** under 100 mM NaCl. The red arrow indicates QTLs detected from six indices.

Supplementary Figure 3 Five differentially expressed genes **(A–E)** for *qRGE60-1-2* and a gene *LOC_Os03g14050* **(F)** for *qRGE60-3-1* are highly expressed in embryos labeled by Geneinvestigator.

Supplementary Table 1 Phenotypic data and information of the 211 rice varieties.

Supplementary Table 2 Criteria for scoring the phenotype of each individual in the population.

Supplementary Table 3 The candidate genes for *qRGE60-1-2* and *qRGE60-3-1*.

Supplementary Table 4 The differentially expressed genes for *qRGE-1-2* and *qRGE-3-1* from the RNA-seq database.
