# Supplementary figures and images for: Genome-Wide Association Study Reveals a Genetic Mechanism of Salt Tolerance Germinability in Rice (Oryza sativa L.)
Source: Front Plant Sci. 2022 Jul 15;13:934515. doi: 10.3389/fpls.2022.934515 (PMC9335074; doi:10.3389/fpls.2022.934515)

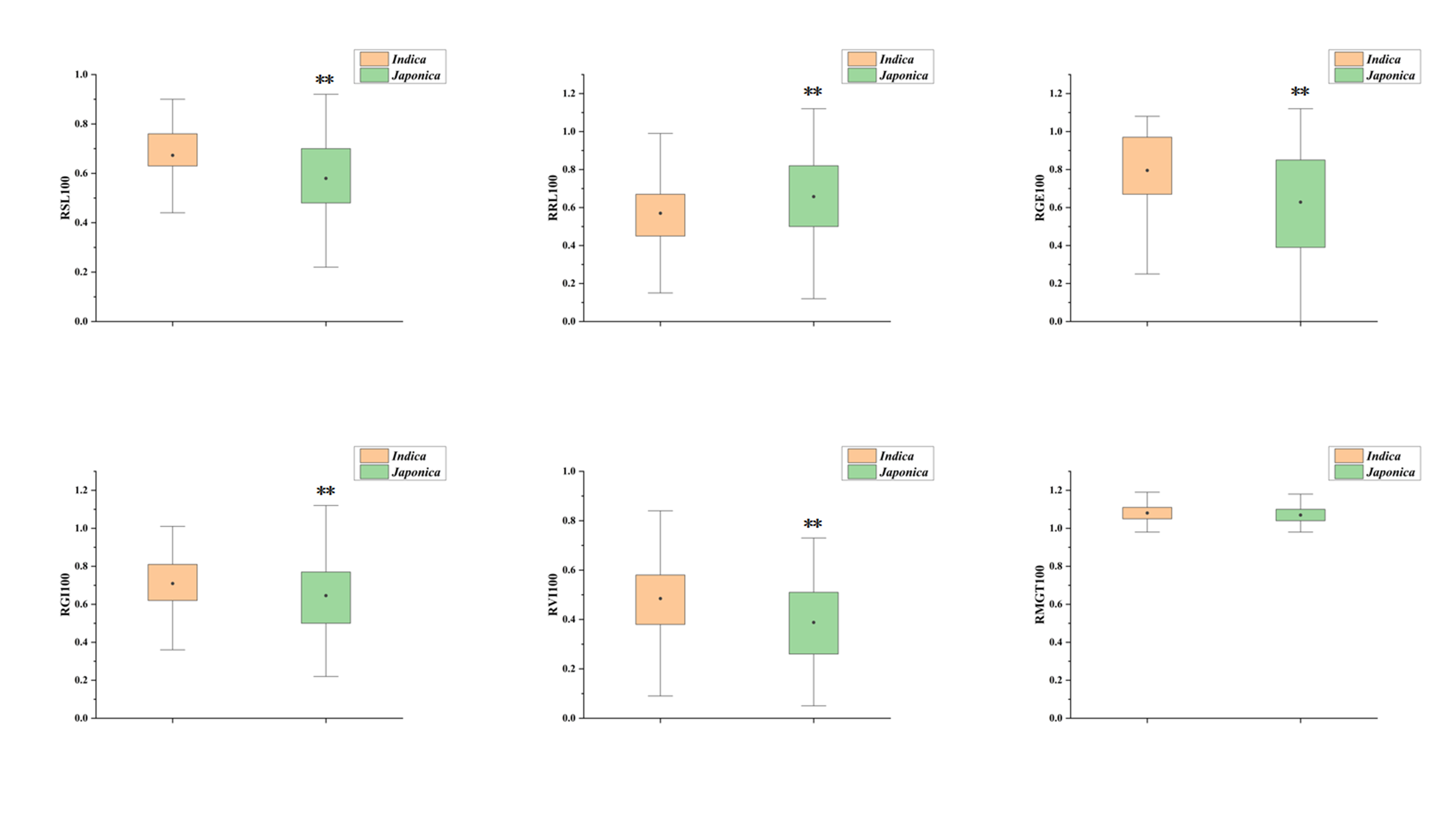

Supplement: Supplementary Figure 1 — Box plot for comparison of six indices between indica and japonica subgroups under 100 mM NaCl. The yellow box represents indica, the green box represents japonica, the black dot in the box represents the median, and the value range of the box is 25–75%, **Indicates significance at the 1% level. [file Image_1.tif]
