# Supplementary material for: Genome-Wide Association Study Reveals a Genetic Mechanism of Salt Tolerance Germinability in Rice (Oryza sativa L.)
Source: Front Plant Sci. 2022 Jul 15;13:934515. doi: 10.3389/fpls.2022.934515 (PMC9335074; doi:10.3389/fpls.2022.934515)
Supplement: Supplementary Figure 2 — Manhattan plots of GWAS for RGI (A), RVI (B), RMGT (C), RRL (D), under 60 mM NaCl and RGE (E), RVI (F), RMGT (G), RSL (H), and RRL (I) under 100 mM NaCl. The red arrow indicates QTLs detected from six indices. [file Data_Sheet_1.docx]

**A**


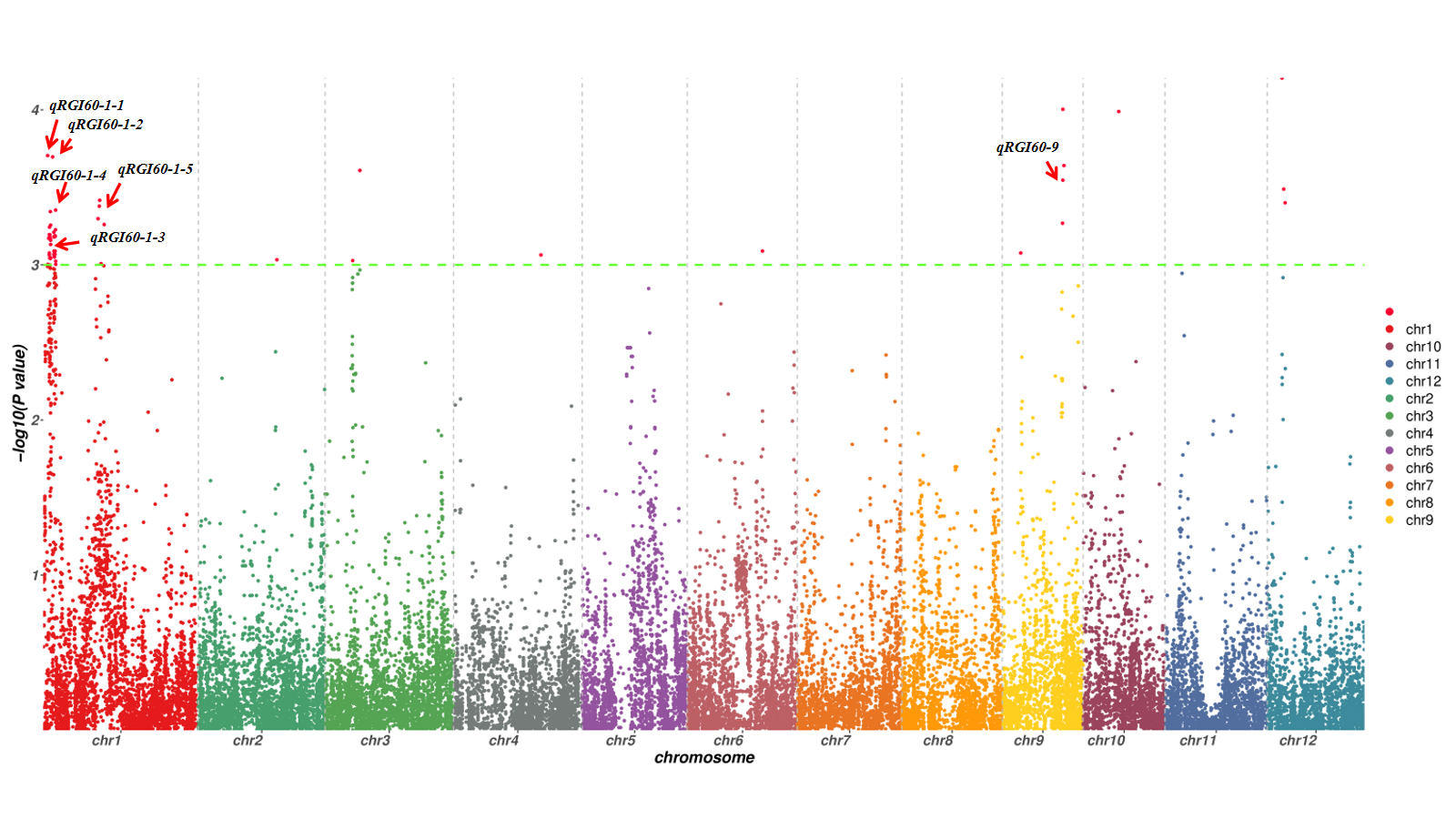


**B**


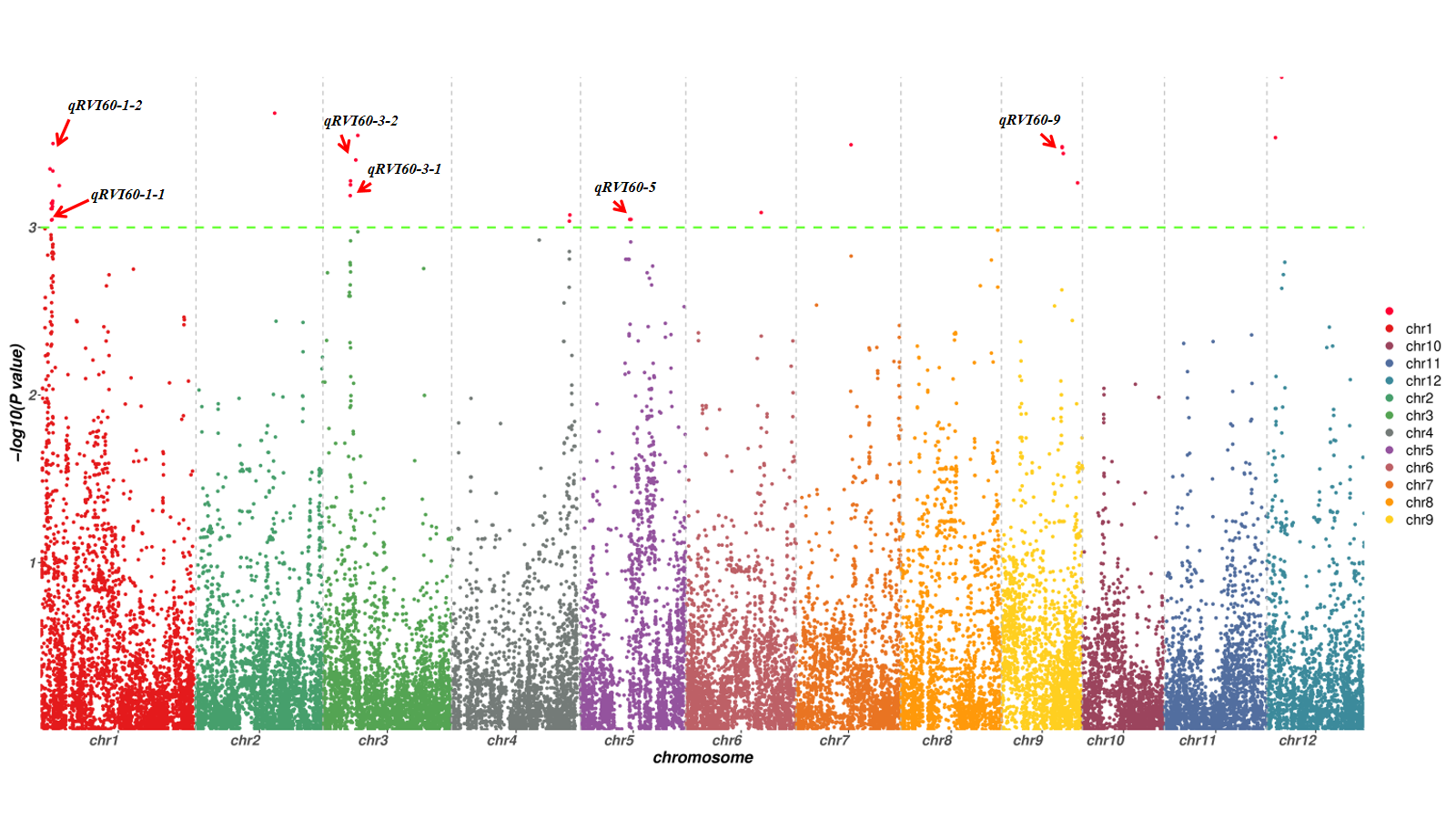


**C**


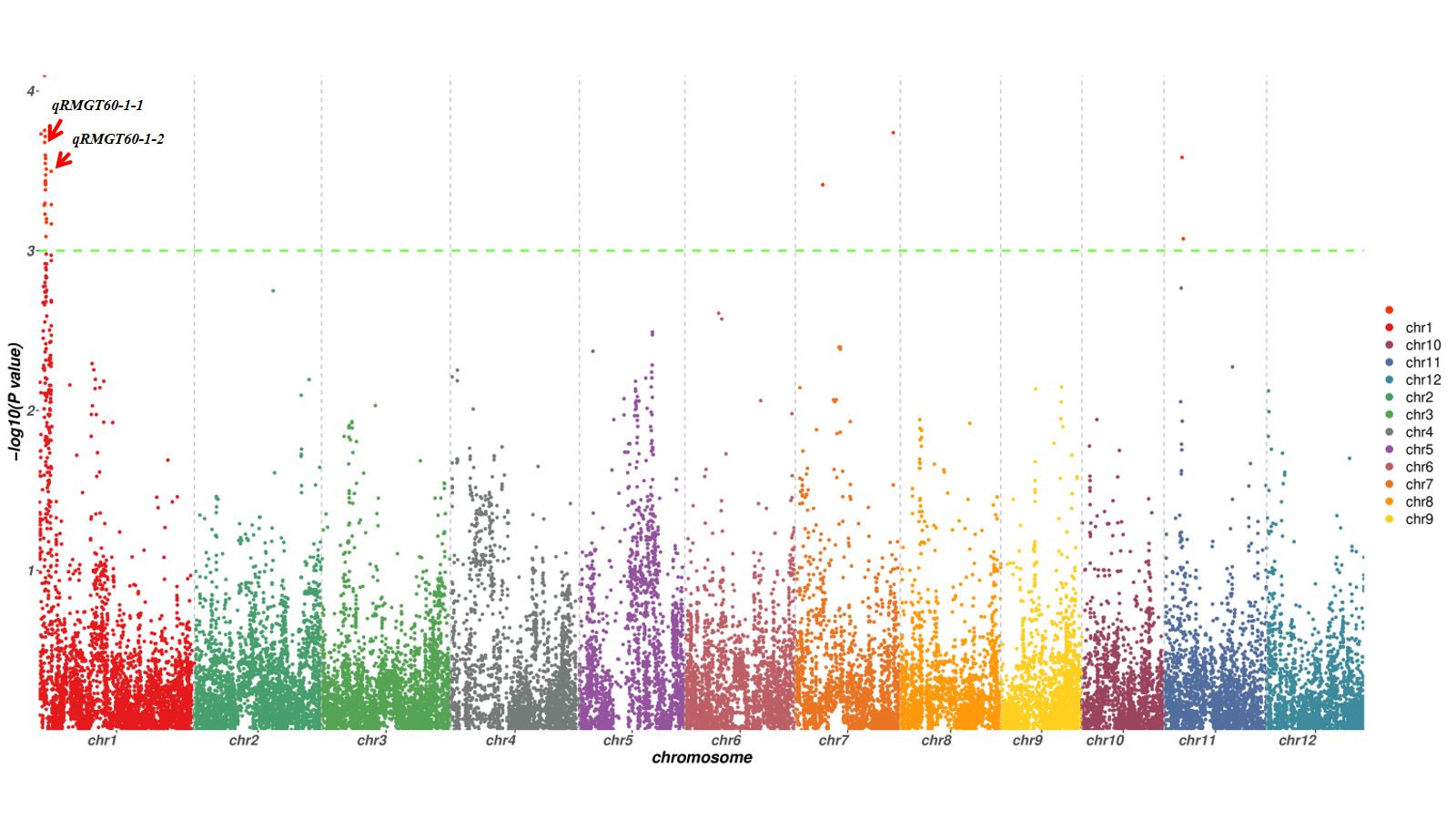


**D**

**
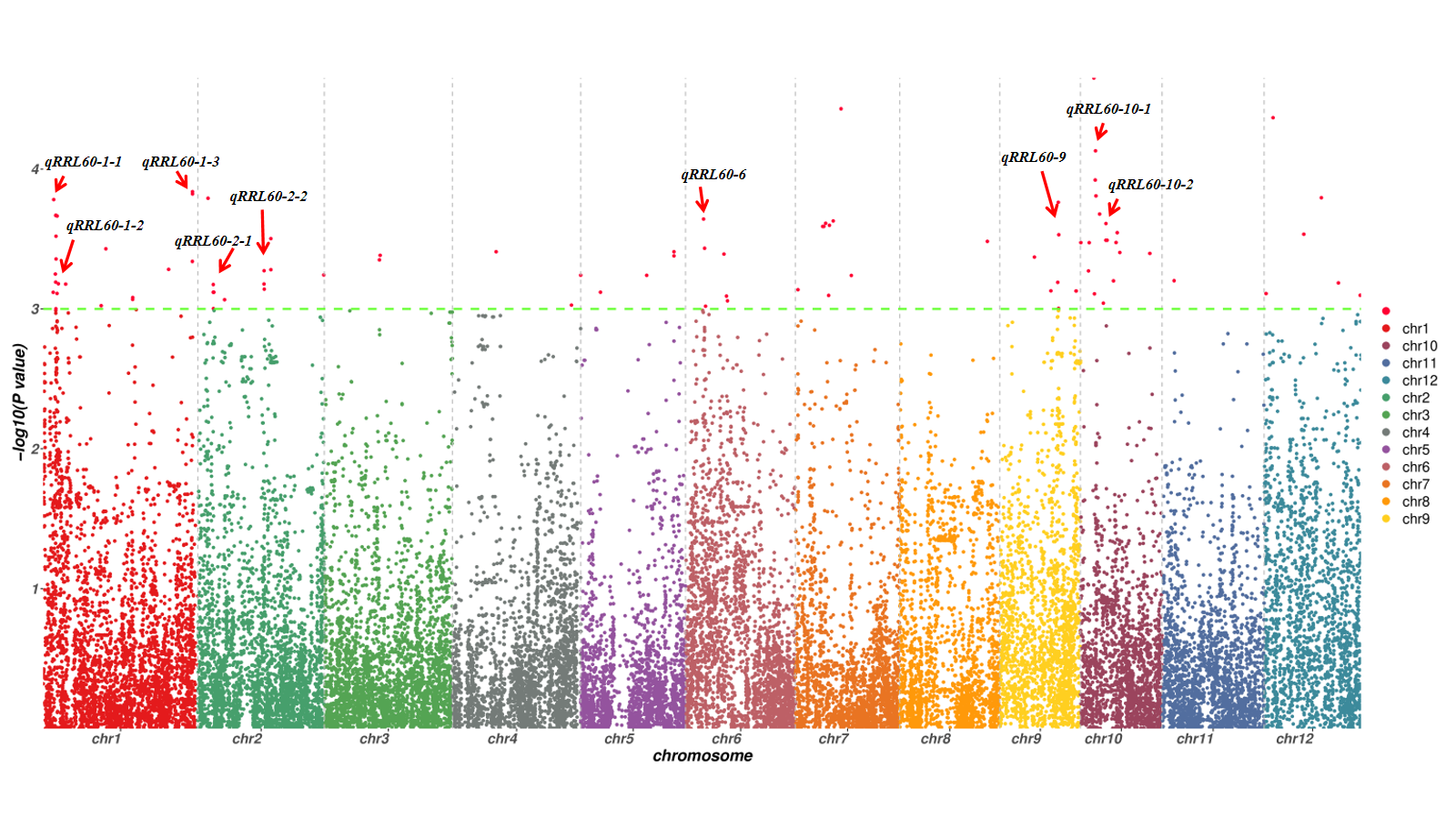
**

**E**

**
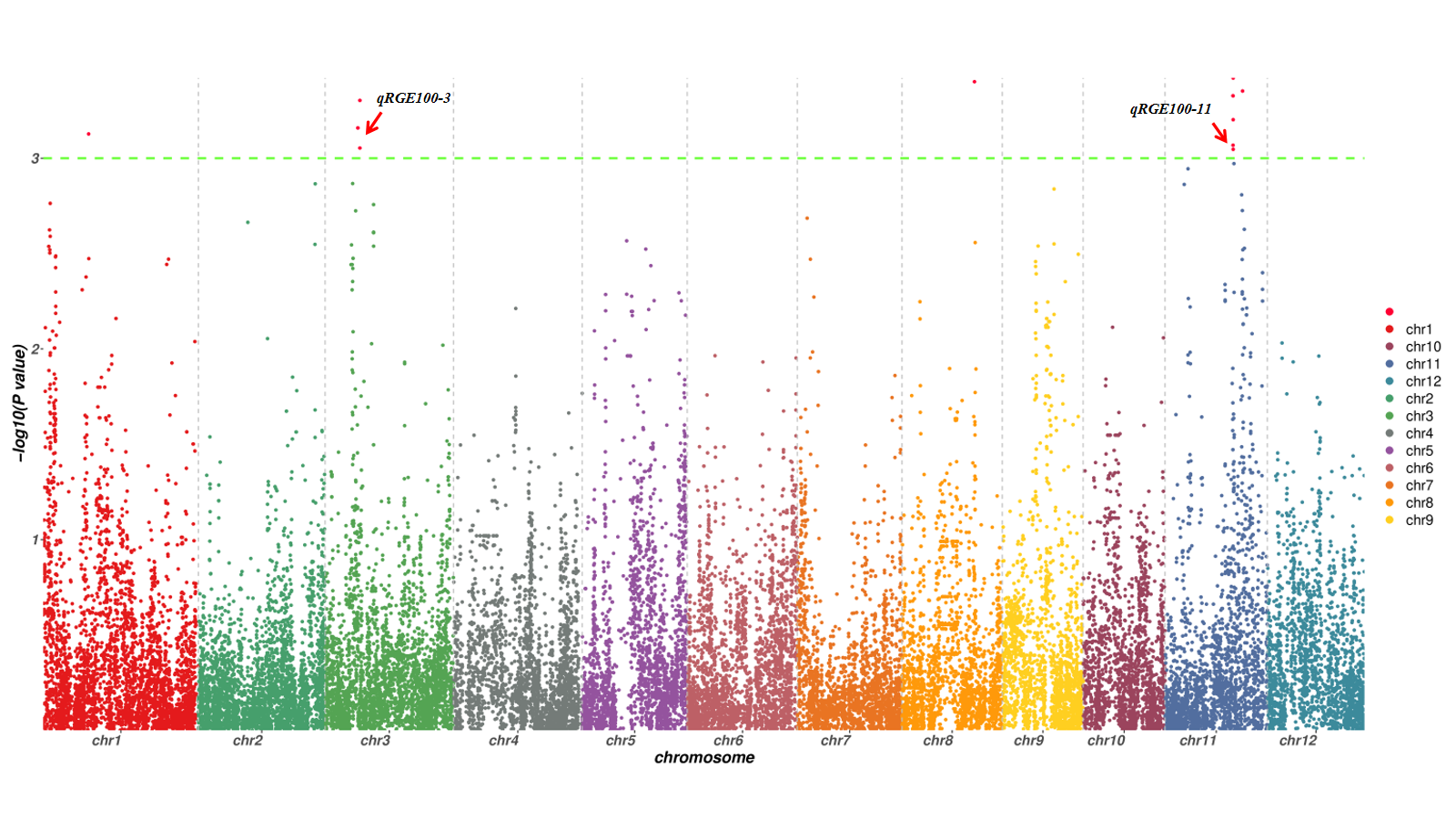
**

**F**

**
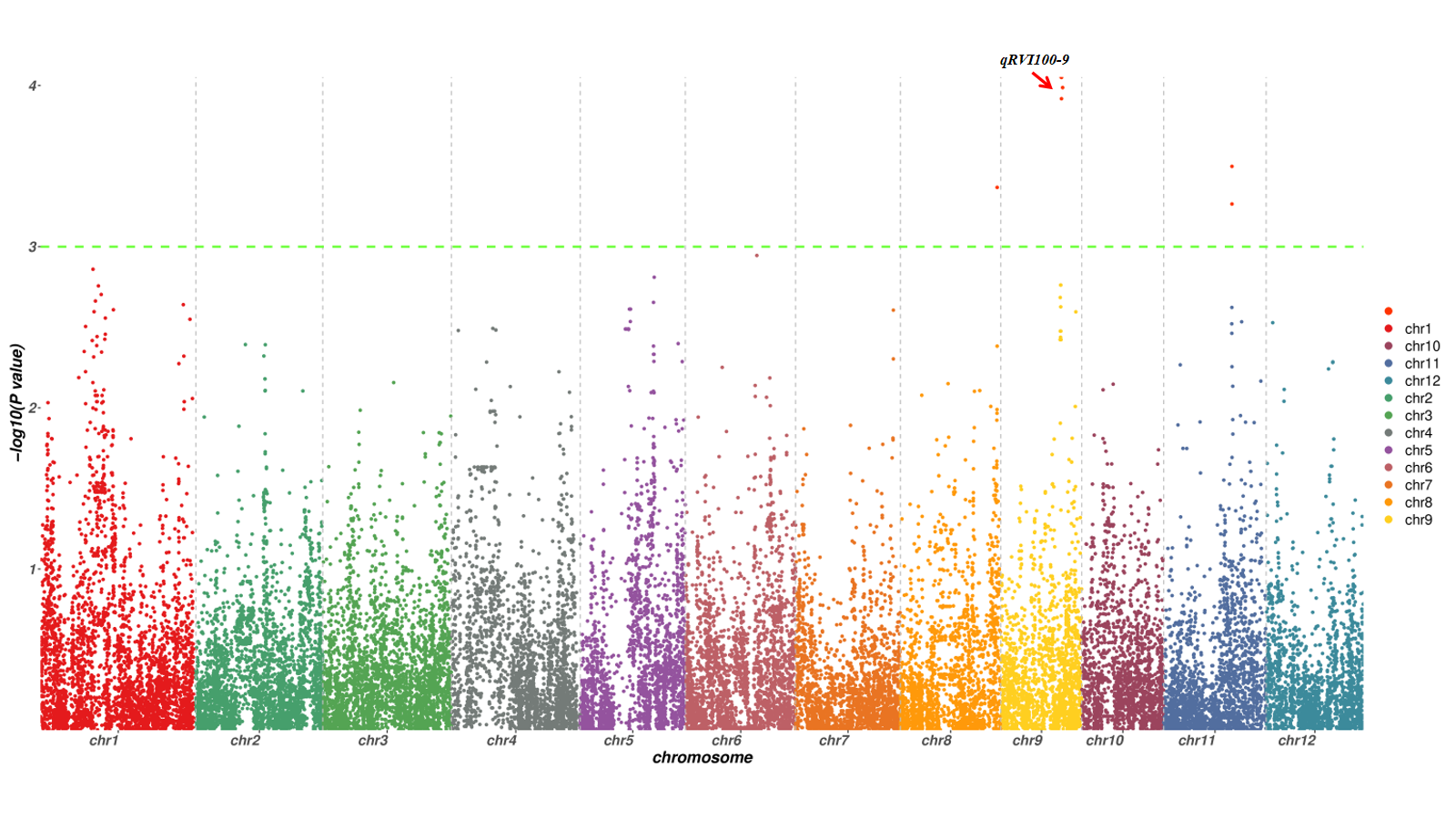
**

**G**

**
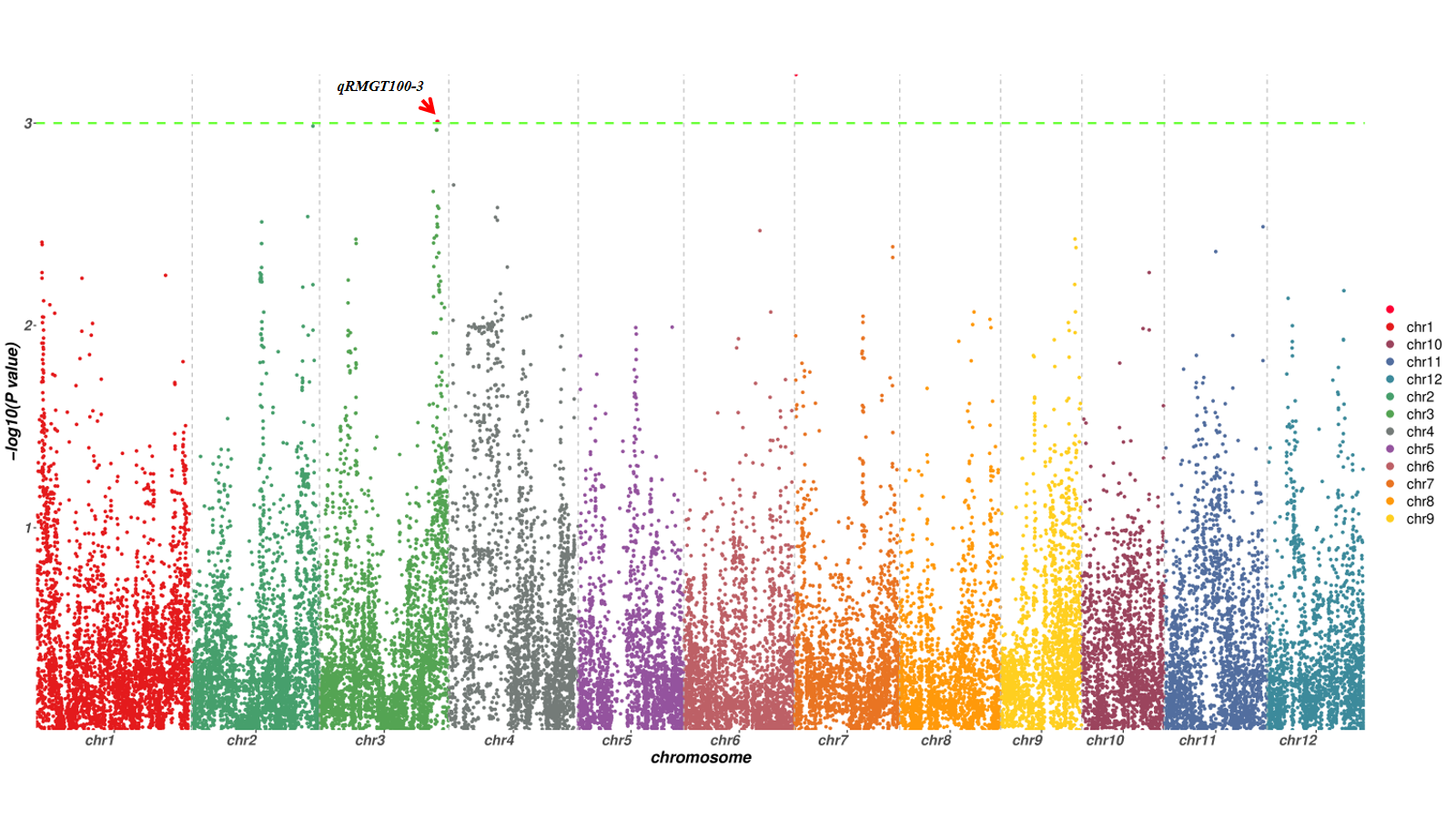
**

**H**

**
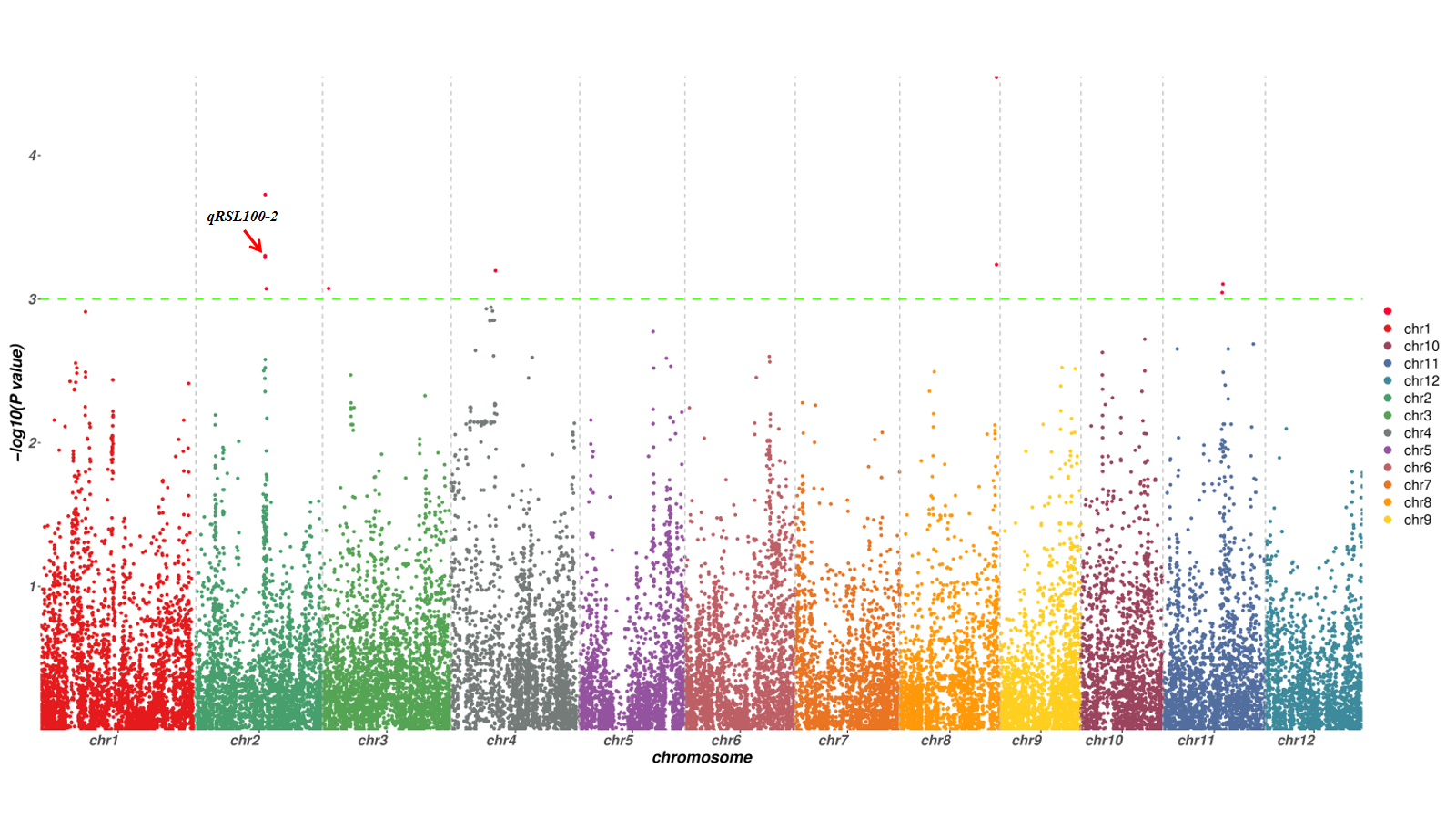
**

**I**

**
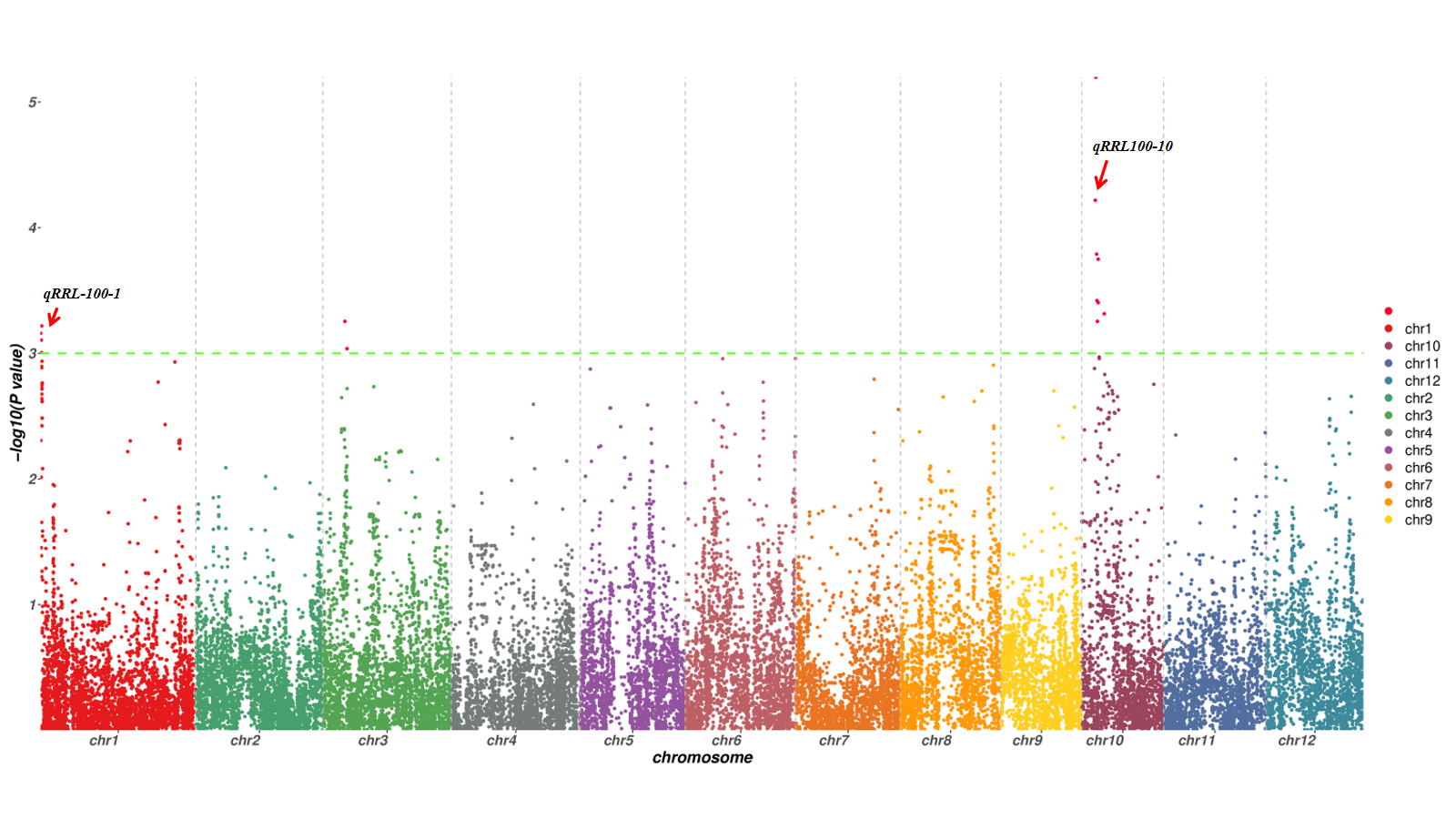
**

**Supplementary Figure 2 |** Manhattan plots of GWAS for RGI (**A**), RVI (**B**), RMGT (**C**), RRL (**D**) under 60 mM NaCl and RGE (**E**), RVI (**F**), RMGT (**G**), RSL (**H**), RRL (**I**) under 100 mM NaCl. The red arrow indicates QTLs detected from six indices.
