# Supplementary material for: Genome-Wide Association Study Reveals a Genetic Mechanism of Salt Tolerance Germinability in Rice (Oryza sativa L.)
Source: Front Plant Sci. 2022 Jul 15;13:934515. doi: 10.3389/fpls.2022.934515 (PMC9335074; doi:10.3389/fpls.2022.934515)
Supplement: Supplementary Figure 3 — Five differentially expressed genes (A–E) for qRGE60-1-2 and a gene LOC_Os03g14050 (F) for qRGE60-3-1 are highly expressed in embryos labeled by Geneinvestigator. [file Data_Sheet_2.docx]

**A B**


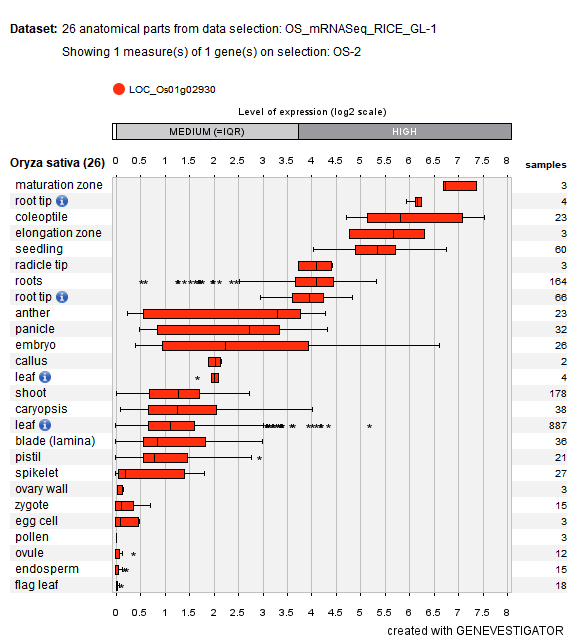

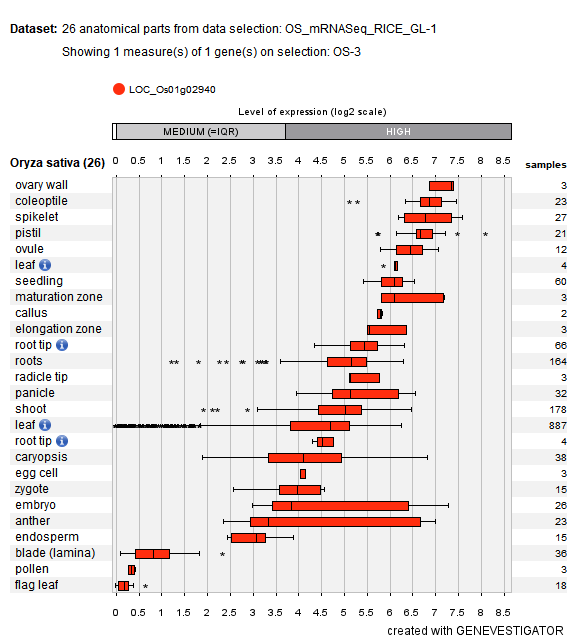


**C D**


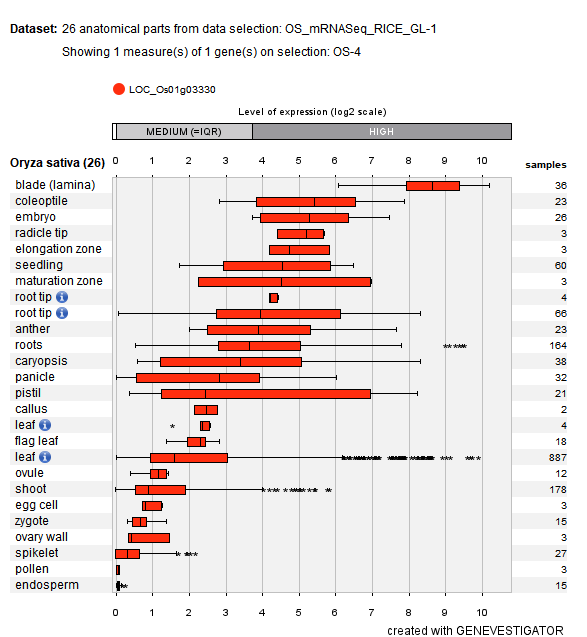

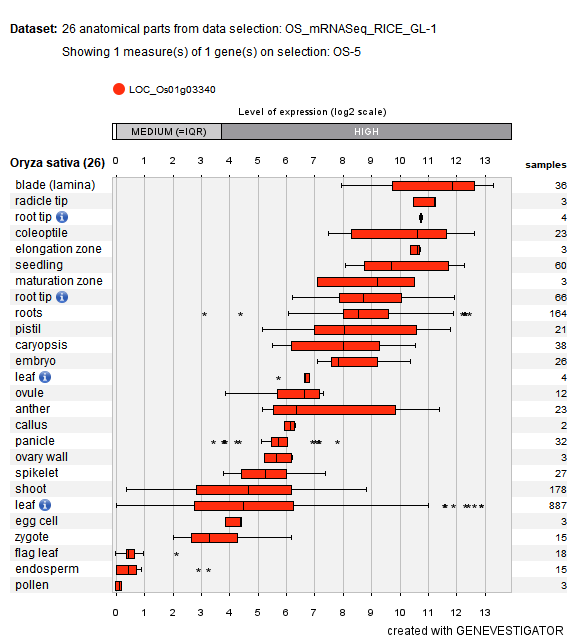


**E F**


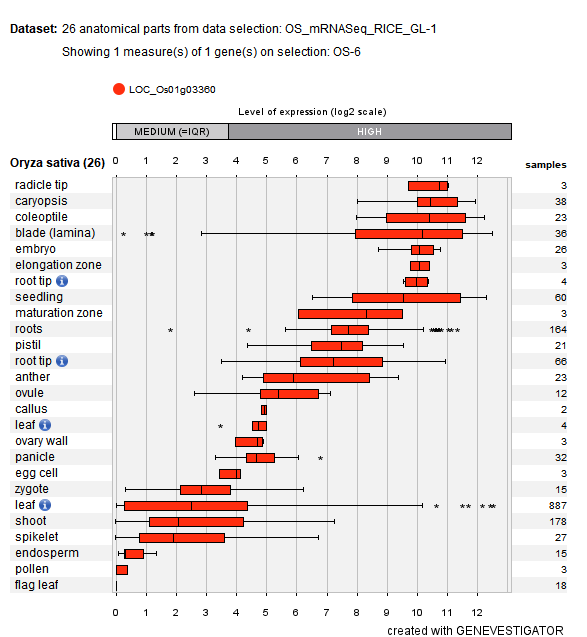

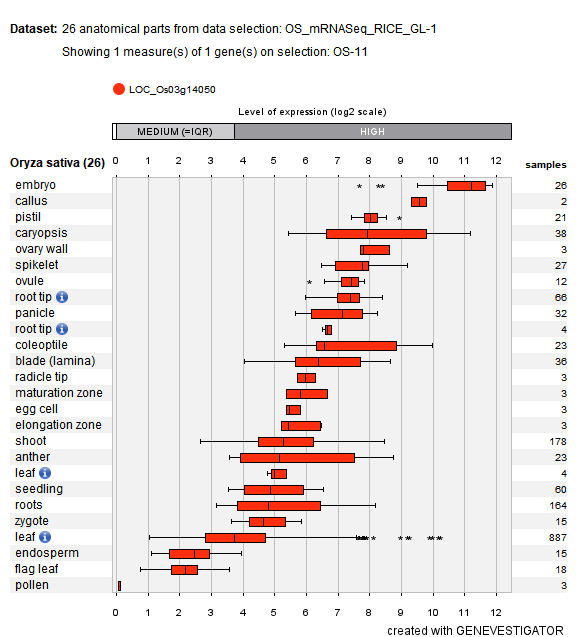


**Supplementary Figure 3 |** Five differentially expressed genes (**A-E**) for *qRGE60-1-2* and a gene *LOC_Os03g14050* (**F**) for *qRGE60-3-1* are highly expressed in embryos labeled by Geneinvestigator.
